# In silico design of a multi-epitope vaccine targeting DENV-1 and DENV-3

**DOI:** 10.1038/s41598-026-35678-0

**Published:** 2026-01-16

**Authors:** Deepthi Ishwar, Shruthi Padavu, Manish Kumar, Pavan Gollapalli, Krishna Kumar Ballamoole, Anoop Kumar, Praveen Rai

**Affiliations:** 1 Department of Infectious Diseases and Microbial Genomics, Nitte University Centre for Science Education and Research (NUCSER), Nitte (Deemed to be University), Paneer campus, Kotekar-Beeri Road, Deralakatte, Mangaluru, 575018 Karnataka India; 2 Department of Bioinformatics and Biostatistics, Nitte University Centre for Science Education and Research (NUCSER), Nitte (Deemed to be University), , Paneer campus, Deralakatte, Mangaluru, 575018 Karnataka India; 3 Molecular Diagnostics Laboratory, National Institute of Biologicals (NIB), Ministry of Health & Family Welfare, Noida, 201309 India

**Keywords:** Dengue virus, Co-infection, Epitope, Immune simulation, Linkers, Multi-epitope vaccine, Virology, Computational biology and bioinformatics

## Abstract

The co-infection of DENV-1 and DENV-3 during endemic outbreaks can be potentially fatal and complicate the diagnostic process. In our study, we have focused on the development of a multiepitope vaccine against DENV-1 and DENV-3 co-infection, utilizing non-structural protein 1 (NS1) and envelope protein (E) as key antigens. B cell and T cell epitopes were predicted for their immunogenicity, antigenicity, and ability to elicit an IFN-γ response. The final construct showed predicted stability (Instability Index: 30.63), antigenicity (0.5509), non-allergenicity, and hydrophilic character (GRAVY: −0.226) based on computational assessments. Tertiary structural validation revealed 90.1% of residues in a favoured region. Molecular docking revealed a stronger binding of the DENV-TLR3 complex. The receptor and vaccine have stable interactions, according to molecular dynamics simulations and free energy estimations (-90 kJ/mol). Strong B and T cell memory responses were demonstrated by immune simulations, accompanied by increased levels of IgG, IFN-γ, and TGF-β. The codon-optimized sequence was successfully cloned into the pcDNA™3.1/V5-His-TOPO^®^ expression vector for potential experimental validation. As a result of this in silico approach, a targeted vaccine for DENV-1 and DENV-3 co-infections is possible, which merits further experimental evaluation.

**Supplementary Information:**

The online version contains supplementary material available at 10.1038/s41598-026-35678-0.

## Introduction

Dengue is a highly infectious viral disease that poses a significant risk to an individual’s life. The global prevalence of dengue fever has risen dramatically. Dengue is an arboviral epidemic disease that is causing a major public health issue and has become an annual epidemic in many parts of Southeast Asia. The disease is widespread throughout 100 nations in the World Health Organization (WHO) regions of the Americas, Africa, Southeast Asia, the Eastern Mediterranean, and the Western Pacific. Asia alone accounts for around 70% of the global illness burden^1^. The European Centre for Disease Prevention and Control (ECDC) estimates that as of December 2023, 86 countries and territories worldwide have reported over 5 million cases and 5000 dengue-related deaths. In India, 94,198 cases with 91 deaths have been reported, according to the National Centre for Vector-Borne Diseases Control (NCVBDC). Unplanned urbanization, climatic change, population immunological factors, and host-pathogen interactions have been attributed to the spread of dengue fever in India^2,3^.

Dengue is a viral infection caused by Dengue Virus (DENV) and was first discovered in 1943 by Ren Kimura and Susumu Hotta^4^. It is transmitted through the bite of infected mosquitoes named *Aedes aegypti* and, to a lesser extent, *Aedes albopictus*, which makes dengue an arboviral disease. DENV belongs to the family Flaviviridae and the genus Flavivirus. The Flaviviridae family also includes other viruses such as the Yellow Fever virus, Zika virus, West Nile virus, Japanese encephalitis virus, and Tick-Borne encephalitis virus. Four antigenically distinct circulating dengue serotypes exist viz., DENV-1, DENV-2, DENV-3, and DENV-4^5^. Dengue fever caused by any of the serotypes can range in intensity from mild febrile sickness to life-threatening conditions like Dengue Haemorrhagic Fever (DHF) and Dengue Shock Syndrome (DSS). The DENV infection is associated with headache, vomiting, nausea, arthralgia, myalgia, swollen glands, and rash (maculopapular or petechial). Plasma leakage and fluid loss are all symptoms of severe dengue, which can lead to hypovolemic shock and multi-organ failure^6^. The symptoms shown by dengue-infected patients are not unique, and the chances of getting confused with other diseases, such as Typhoid fever, chikungunya, malaria, etc., are high. Diagnosis and treatments based only on clinical symptoms are unreliable^7^.

To develop effective treatments or potential cures, it is necessary to have a comprehensive understanding of the structural composition of the Dengue virus. DENV is an enveloped, single-stranded positive-sense RNA containing 10,723 nucleotides, of which 10,173 nucleotides are accountable for the single open reading frame that codes for a polypeptide with 3,391 amino acid residues. Dengue virus genome encodes three structural proteins [envelope (E), pre-membrane (PrM), and capsid (C)] and seven non-structural proteins (NS1, NS2A, NS2B, NS3, NS4A, NS4B, NS5). This genome is packed inside the icosahedral-shaped nucleocapsid^8^. Structural and non-structural proteins of DENV play a role in virus replication in the host. The DENV serotypes can be subdivided into several genotypes based on envelope genes, providing humans with partial cross-protective immunity against the other serotypes^5,9^.

NS1 is the most critical non-structural protein in dengue virus pathogenesis and is a highly conserved glycoprotein among all serotypes. The NS1 protein is composed of 352 amino acid residues. They share sequence similarity of approximately 70% with all four DENV serotypes and 40–50% with other flaviviruses. NS1 levels are very high during the acute phase of the disease^10^. NS1 is a vital protein for viral replication and pathogenicity. NS1 is a multifunctional glycoprotein. It is initially formed as a monomer and forms a dimer after the post-translational modification in the lumen of the endoplasmic reticulum as a membrane-associated protein. They are then released into the blood circulation as a secretory protein in a hexameric form. Furthermore, they activate the cytokine production from innate immune cells via binding to Toll-like receptor (TLR)−4^11^. Envelope proteins (E) are the major targets for the host immune system since they are the first proteins to interact with the host. E antigen is primarily responsible for the interaction and subsequent invasion of host cells^12^. The E protein has three domains, Domain I (DI), Domain II (DII), and Domain III (DIII). The epitope of the virus determines neutralization and Antibody-Dependent Enhancement (ADE). At one end of the protein molecule, DIII is responsible for the virus gaining entry into the host cell. While DII is present at the other end, it helps in the fusion of the viral membrane with the endosomal membrane to release the viral genome into the host cell^13^. Thus, NS1 and E proteins have been the main targets for producing neutralizing antibodies, and thus, the focus is on designing vaccines.

During primary infection, the viremia level in a host is suppressed by neutralization. Still, in secondary heterotypic serotype infection, ADE occurs due to pre-existing neutralizing antibodies, which increase viremia, a major immunopathological challenge that complicates vaccine design^14^. During dengue pathogenesis, cross-reactive immune responses emerge not only among flaviviruses but also between the dengue serotypes themselves, making it more challenging to design effective vaccines and provide immune protection. In some dengue-endemic areas, co-infection may occur due to the increased co-circulation of serotypes^15^. Though DENV-2 is more prevalent, the rise in DENV-3 was detected in 2021 and 2023. Research on the evolution of the Dengue virus in endemic areas is also being done as spontaneous mutations might result in changes in serotypes or genotypic variants, which can trigger new outbreaks. A few reports of DENV-1 and DENV-3 co-infection outbreaks have also been found^16–19^. Dengue fever is usually treated symptomatically, but there is no safe or effective vaccine against dengue. This increasing need for treatment for the public led to the development of vaccines.

The first licensed vaccine is Dengvaxia^®^ (CYD-TDV – developed by Sanofi Pasteur), a live attenuated tetravalent vaccine. The second recently licensed vaccine in Indonesia is QDENGA^®^ (TAK-003 – developed by Takeda Pharmaceuticals), which is also a live attenuated tetravalent vaccine. Though the efficacy was 80.2% for the initial 11 months, it was reduced in the subsequent studies based on DENV serotypes. Seronegative individuals had a higher hospitalisation rate, and their safety outcomes were comparable to those of Dengvaxia^®^^20^. Recent evaluations confirm that licensed dengue vaccines show serotype-dependent efficacy and elicit the most robust protection in individuals with prior dengue exposure, leaving a protective gap in seronegative populations and across geographic regions^21^. Other potential vaccines are undergoing clinical trials in different phases, such as TDEN-LAV (WRAIR/GSK), Live-attenuated, TDENV-PIV (WRAIR/FioCruz/GSK), Inactivated adjuvanted, D1ME 100/TVDV (NMRC), DNA vaccine, V180 (DEN-80E) (Merck/NIAD), Recombinant (subunit), and DENV-1-LVHC live-attenuated. However, each mentioned vaccine has its drawbacks, and some data are still pending^22^. Several treatment techniques, including immunometabolic modulations of glycolysis, lipid metabolism, and signalling cascades, have been found to inhibit dengue virus replication; however, they are still in early phases of validation. Despite the mentioned advances, approaches involving vaccine development are still important to prevent the infection, particularly in the endemic areas, involving the co-circulation of multiple serotypes^15^. The cases of DENV-1 and DENV-3 co-infection are associated with increased severity^23^. This co-infection underscores the challenge in clinical management and highlights the need for early detection and vigilant monitoring in regions where multiple serotypes circulate concurrently.

Currently, dengue-infected patients are being treated symptomatically. The vaccines provide partial and inconsistent protection among serotypes, and the co-infection cases have complicated the diagnostic process. The DENV-1 and DENV-3 co-infection cases have been reported in the endemic regions, making it potentially fatal. However, no prior work has developed a multiepitope vaccine against the co-infection of DENV-1 and DENV-3. An in silico study has paved the way for constructing a vaccine using a computational approach, which is cost-effective compared to conventional studies. The purpose of this study was to identify highly conserved and immunogenic epitopes shared by DENV-1 and DENV-3, and to develop a multiepitope vaccine targeting these epitopes. Using this approach, it is possible to create a safe vaccine, leading to a new paradigm for combating dengue co-infection.

## Results

### Analysis of protein structure

**Fig. 1:**
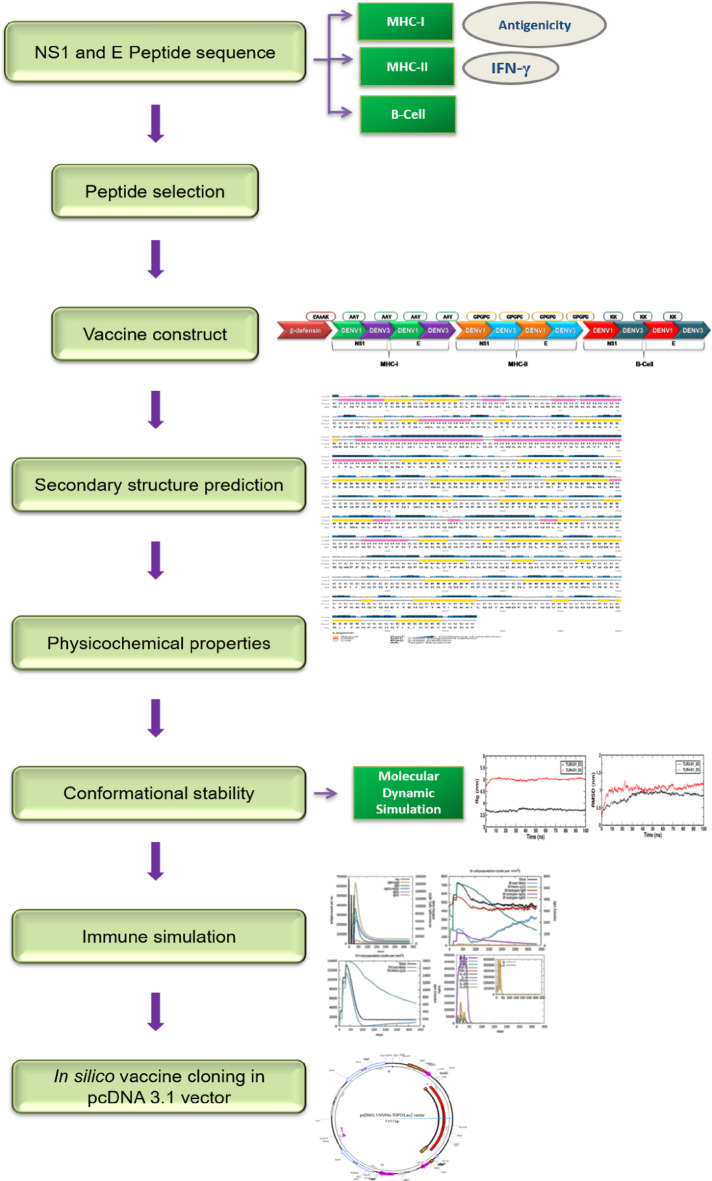
Overview of the multi-epitope vaccine design study against DENV-1 and DENV-3.

To develop a multi-epitope vaccine, NS1 and E protein sequences were retrieved from the NCBI database. The NS1 and E proteins of DENV-1 & −3 contain 352 and 495 amino acid residues, respectively. ProtParam server was used to estimate the amino acid composition of NS1 and E protein. The molecular weight of NS1 and E protein of DENV-1 is 40.03 kDa and 53.86 kDa, and DENV-3 is 39.83 kDa and 53.69 kDa, respectively. The amino acid composition of NS1 and E proteins of DENV-1 & −3 is given in Supplementary Tables 1 & 2. With the help of these amino acid sequences, cytotoxic T lymphocytes (CTL) and helper T lymphocytes (HTL) epitopes were further examined. The general process of the vaccine design methodology is illustrated in Fig. 1.

### T and B cell epitopes prediction and selection

An HLA allele reference set and the IEDB-recommended 2020.09 NetMHCPan EL 4.1 were used to select MHC I epitopes of length 9-mer. For both the NS1 and E proteins of DENV-1 & −3, the best strong binding affinity peptides were chosen based on percentile rank. A peptide’s ability to bind to a large number of alleles improves with decreasing percentile rank. Therefore, a percentile rank of less than 0.5 was chosen, which roughly translates to an IC50 value of 50 nM. Using the IEDB server, the peptides were further selected based on their positive immunogenic ratings. To anticipate their antigenicity, the chosen peptides were sent to the VaxiJen server. A total of 16 and 31 NS1 and E protein peptides from DENV-1 and 14 and 36 NS1 and E protein peptides from DENV-3 were chosen (Supplementary Tables 3 & 4). IEDB suggested the 2.22 technique using an HLA allele reference set and anticipated epitopes for MHC II. Additionally, MHC II epitopes with a 15-mer length were chosen using the IEDB server. Each peptide’s anticipated percentile rank in the server was chosen up to 2.5, or about 250 nM. Better binding capacity is indicated by a lower percentile rank. The chosen peptides’ IFN-γ score was further examined. Potential peptides were just those whose IFN-γ values were positive. A total of 30 and 36 NS1 and E protein peptides from DENV-1 and 14 and 30 peptides from DENV-3 were chosen (Supplementary Tables 5 and 6). For both the NS1 and E proteins of DENV-1 & −3, linear epitopes with short amino acid lengths, or 9-mers, were predicted using the ABCPred service. The DENV-1 NS1 protein has 39 predicted epitopes, while the DENV-3 NS1 protein has 35 predicted epitopes, with corresponding prediction scores of 0.96 and 0.94. The DENV-1 E protein was projected to have 54 epitopes, while the DENV-3 E protein was given 51. Their initial prediction scores were 0.92 and 0.94, respectively (Supplementary Tables 7 and 8). The docking results obtained from the PatchDock server revealed stable and specific interactions between the selected epitopes and their corresponding HLA molecules (Supplementary Figure S1).

### Multi-epitope vaccine construction

**Fig. 2:**
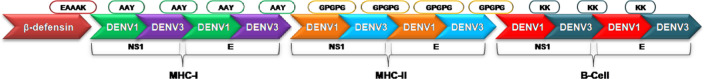
Schematic representation of the constructed multi-epitope vaccine against DENV-1 and DENV-3.

A multi-epitope vaccination against DENV was created using specific epitopes from MHC I, MHC II, and B cells (Table 1). Only four and three peptides from the DENV-1 and DENV-3 NS1 proteins, respectively, and three and four peptides from the DENV-1 and DENV-3 E proteins, respectively, were chosen under MHC I epitopes (CTL). On the other hand, two B cell epitopes and three MHC II (HTL) epitopes were chosen from the DENV-1 and − 3 NS1 and E proteins, respectively. Eight B cell epitopes, twelve HTL, and fourteen CTL epitopes were chosen. Linkers AAY, GPGPG, and KK were used to join these epitopes, respectively (Fig. 2). A beta-defensin adjuvant was linked to the vaccine’s N-terminal using the linker EAAAK in order to elicit the immune response and stop it from interacting with the vaccine that was created. The vaccine’s design contains 575 amino acids in total.

**Table 1.**
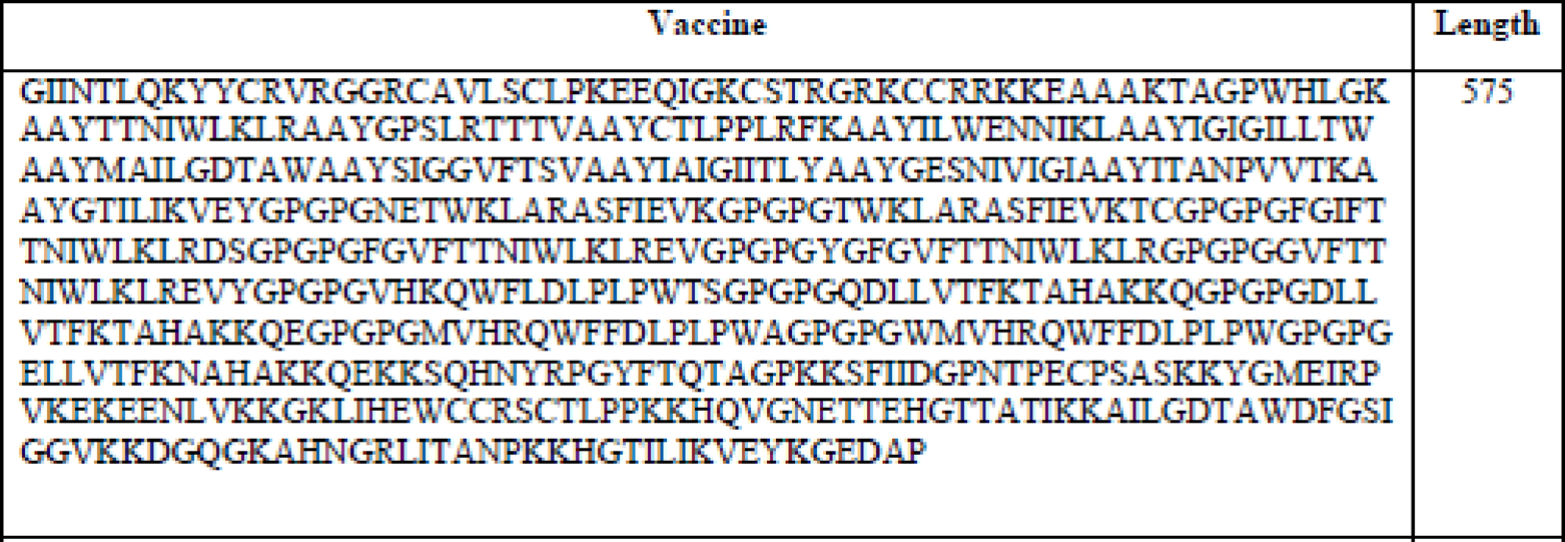
Constructed multi-epitope vaccine.

### Secondary structure prediction of vaccine

**Fig. 3:**
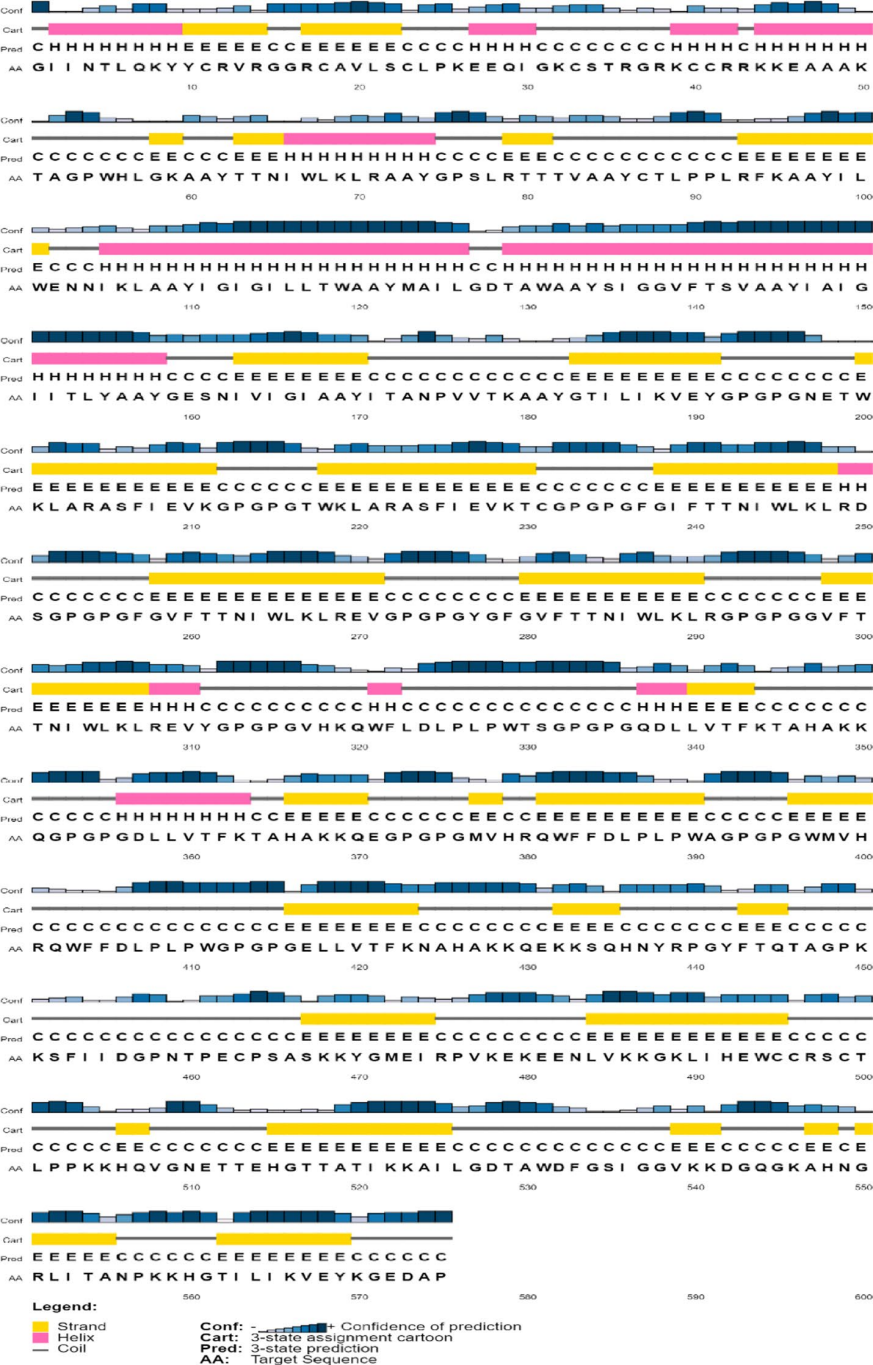
Secondary structure of the constructed multi-epitope vaccine.

The secondary structure of the constructed vaccine was predicted using the PSIPRED server. The designed vaccine comprised an α-helix with 102 amino acids, β-strands with 210 amino acids, and a coil with 263 amino acids. The structural deformities in the constructed vaccine were not found. The secondary structure of the vaccine revealed that 17.73% are α-helix, 36.52% are β-strands, and 45.73% are coil (Fig. 3).

### Allergenicity and physicochemical properties of the vaccine

Based on the ExPASy-ProtParam server, the constructed vaccine comprised 575 amino acids with a molecular weight of 62.63 kDa. The theoretical pI was 9.77. The amino acid composition is given in Table 2. The total number of negatively charged residues (Asp + Glu) is 36, and the number of positively charged residues (Arg + Lys) is 75. The estimated half-life of mammalian reticulocytes (in vitro) is 30 h, while the estimated half-life in yeast and *Escherichia coli* (in vivo) are > 20 h and > 10 h, respectively. The Instability index is 30.63, which classifies the protein as stable. The aliphatic index is 78.61 and the GRAVY is −0.226. The negative value in GRAVY indicates that the vaccine is hydrophilic.

**Table 2:** Amino acid composition of the constructed multi-epitope vaccine.

Amino acids	Residues	Percentage
Ala (A)	50	8.70%
Arg (R)	22	3.80%
Asn (N)	18	3.10%
Asp (D)	12	2.10%
Cys (C)	12	2.10%
Gln (Q)	13	2.30%
Glu (E)	24	4.20%
Gly (G)	74	12.90%
His (H)	13	2.30%
Ile (I)	38	6.60%
Leu (L)	45	7.80%
Lys (K)	53	9.20%
Met (M)	4	0.70%
Phe (F)	22	3.80%
Pro (P)	46	8.00%
Ser (S)	16	2.80%
Thr (T)	45	7.80%
Trp (W)	20	3.50%
Tyr (Y)	21	3.70%
Val (V)	27	4.70%
Pyl (O)	0	0.00%
Sec (U)	0	0.00%

### Tertiary structure prediction, validation, and refinement

**Fig. 4:**
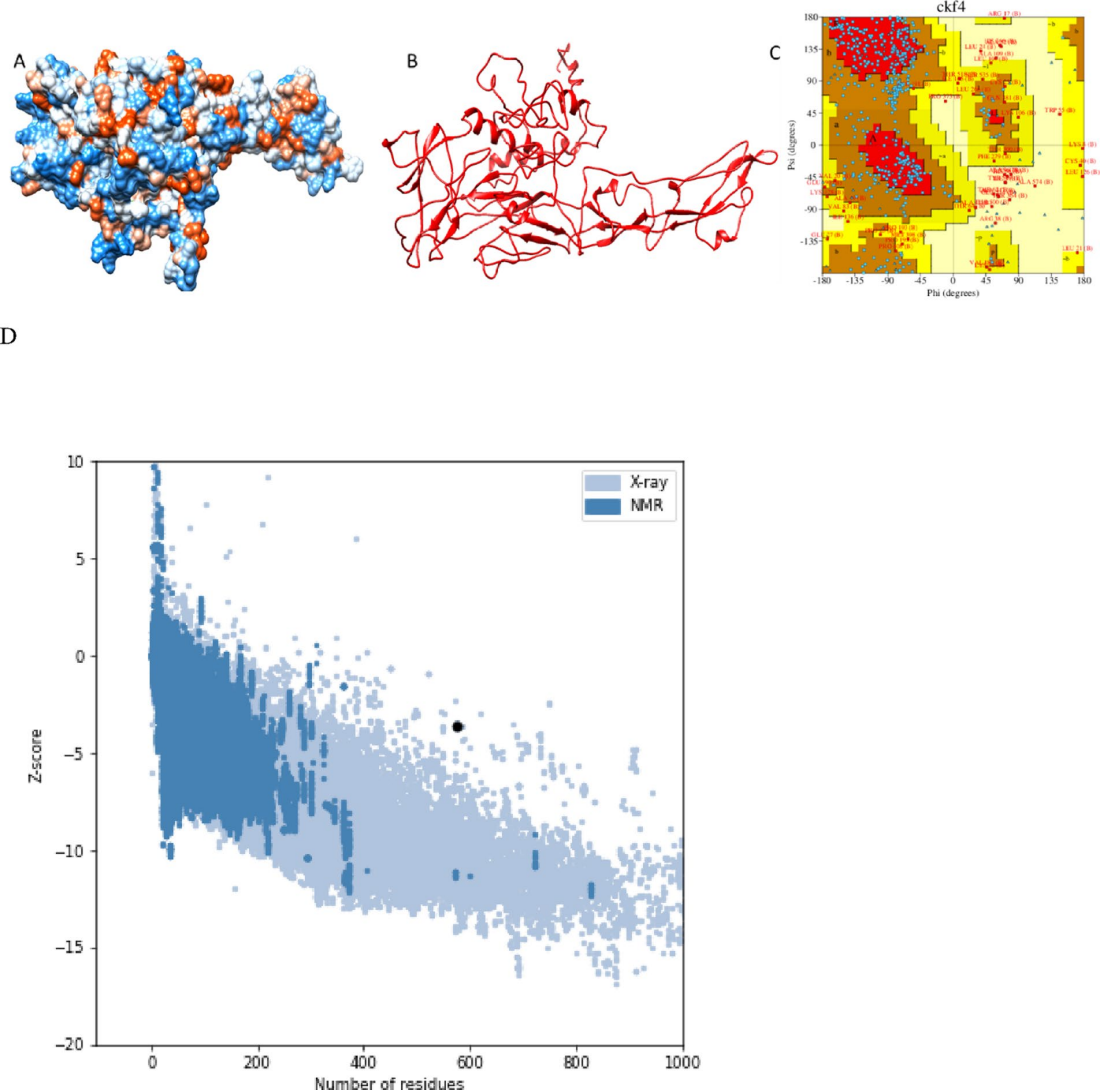
Structure prediction, refinement and validation of the 3D structure of the prepared vaccine construct (**A**) predicted 3 d structure by I-TASSER (**B**) refinement of the 3D structure of the chimeric vaccine by using galaxyRefine server (**C**) PROCHECK’s Ramachandran plot illustrates that the residues are placed in the allowed and favoured regions. (**D**) Z-score (−3.62) is displayed in the 3D structure’s PROSA validation. Overall model quality is shown by the z-score.

I-TASSER projected five models overall using ten threading templates with C-scores; the models were selected based on their high confidence C values, which range from − 0.63 to −4.18. The GalaxyRefine server helped five models to improve the 3D structure of the chimeric vaccine (Fig. 4A and B). The 3D structure was examined in search of likely errors and quality concerns. The Ramachandran plot produced by PROCHECK shows that 5.9% of the residues are in the further permitted areas and 90.1% of the residues are in the areas with most favourable and also permitted (Fig. 4C). Based on its model quality score—which includes GDT-HA (0.9265) and RMSD—0.475—Model 1 was selected for molecular dynamics (MD) simulation, and ProSA-web showed that the model is stable (Fig. 4D).

### Conformational BCEs & disulfide engineering of the vaccine construct

A total of seven conformational (discontinuous) B cell epitopes were predicted in the designed vaccine construct, each exhibiting a predicted score above 0.5, indicating strong antigenic potential and favourable surface accessibility (Supplementary Table 9). Using Disulfide by Design (DbD2), the vaccine construct’s model identifies 90 residue pairs that may potentially create a disulfide bond, as predicted by the Disulfide by Design 2.13 server (Supplementary Table 10). The geometric criteria suggested by Craig et al. (χ³ dihedral angle between − 87° and + 97°; bond energy below 2.2 kcal·mol⁻²), five possible pairs of residues were found to be good candidates for disulfide bond engineering in the modelled protein structure. Here are the expected pairs and the parameters that went with them: Serine 34 joins with Cysteine 40 at − 91.03° and has an energy of 2.01 kcal·mol⁻². Alanine 47 joins with Threonine 51 at − 91.76° and has an energy of 1.00 kcal·mol⁻². Glycine 192 joins with Alanine 223 at − 73.56° and has an energy of 1.45 kcal·mol⁻². Lysine 201 joins with Phenylalanine 225 at − 68.16° and has an energy of 0.97 kcal·mol⁻¹. and Glycine 216 joins with Leucine 220 at + 97.91° and has an energy of 1.04 kcal·mol⁻¹. Three pairs (Gly192–Ala223, Lys201–Phe225, and Gly216–Leu220) strictly met the geometric and energetic criteria. The other two pairs (Ser34–Cys40 and Ala47–Thr51) had small deviations (≤ 5°) from the optimal χ³ range, but these shouldn’t make it too hard to form disulfide bonds after the structure has been optimized. Altogether, these designed pairs of residues make good spots for disulfide bridges to attach, and they are expected to make the modelled protein more stable and rigid through smart stabilization design.

### Molecular Docking of vaccine construct with receptors

**Fig. 5:**
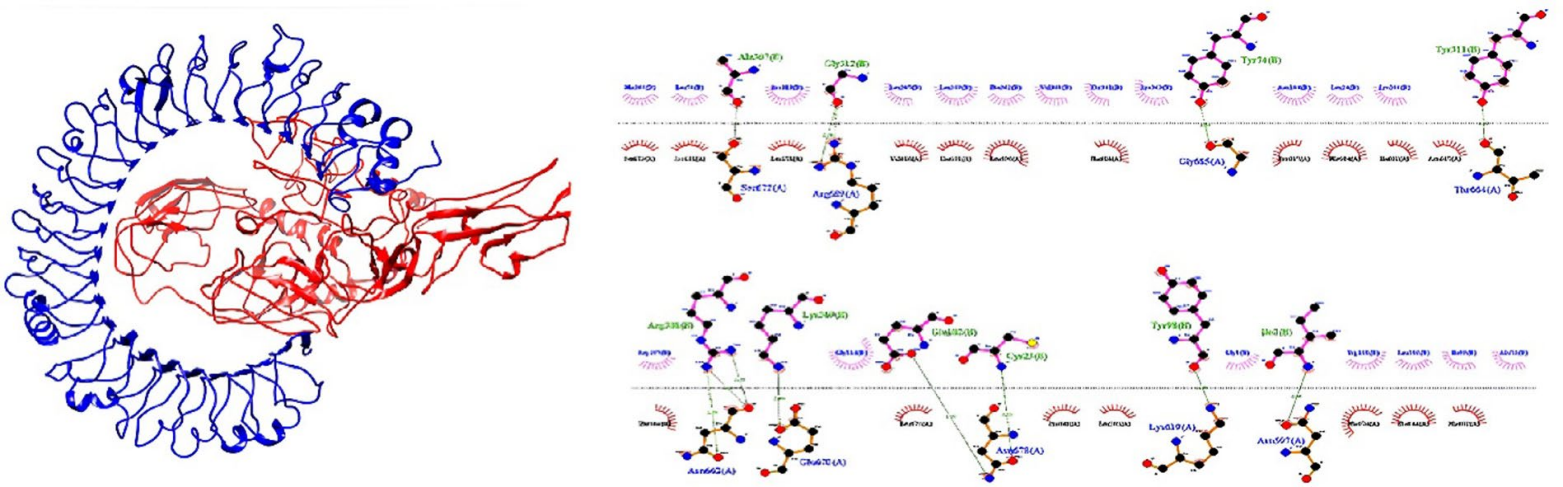
The docked complex of the multi-epitope vaccine and TLR3 was visualized using the UCSF Chimaera software and the interaction in the docked complex has been shown using Ligplot software.

The binding affinity and mode of interaction between a ligand and receptor molecule can be investigated by means of molecular docking. A fundamental human protein for pathogen recognition and immune response is the toll-like receptor. Accordingly, we choose TLR3 as the immunological receptor for molecular docking studies. With the ClusPro 2.0 server, the updated 3D model of our constructed vaccine is docked with the TLR3 immunological receptors. After the docked complex, the clusters with the lowest energy ratings were selected. LigPlot program examined the residuals of the interactions between TLR3 and vaccine construct (Fig. 5).

### Conformational stability analysis of the designed vaccines

**Fig. 6:**
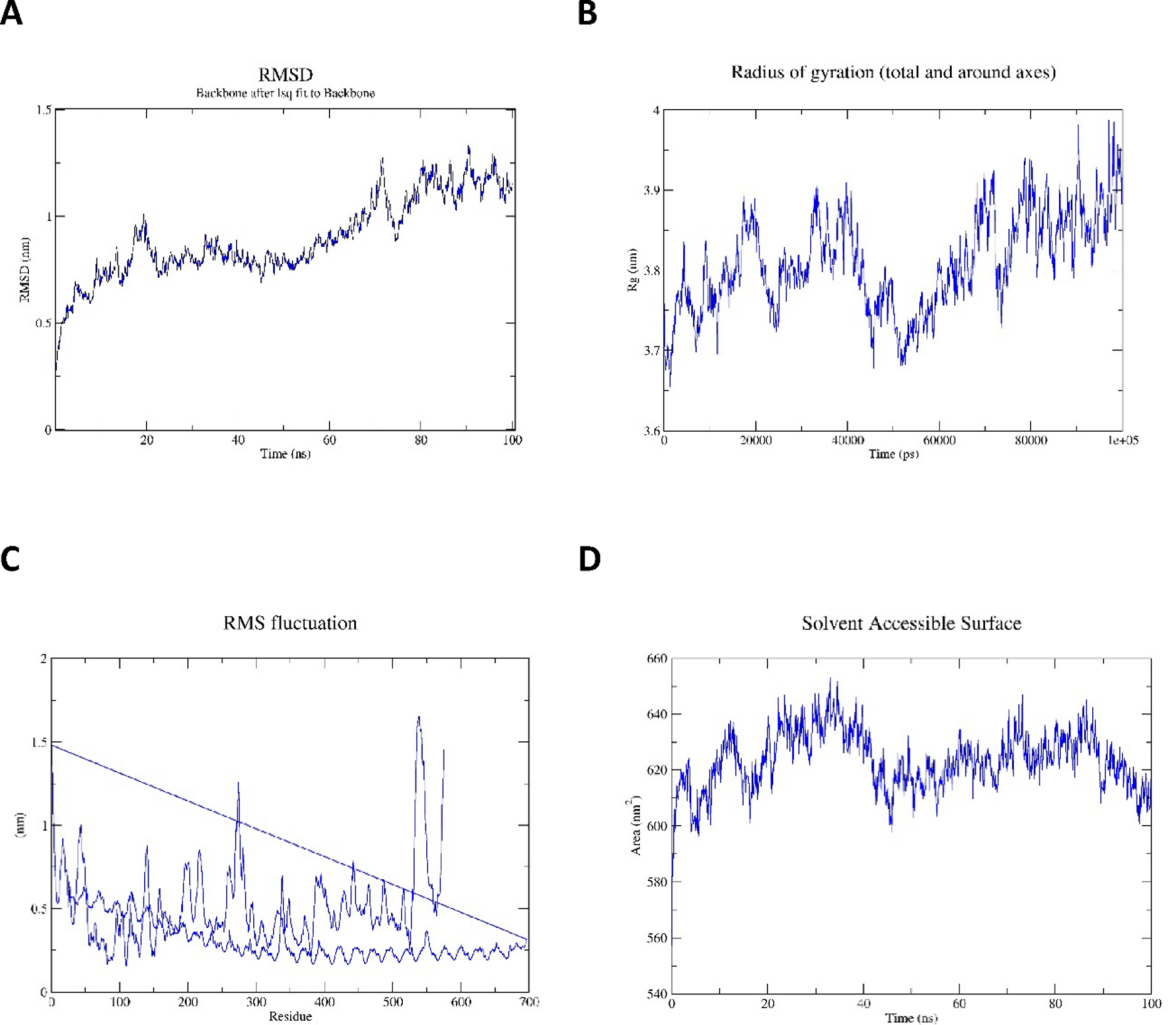
Molecular dynamic simulation of (**A**) Root Mean Square Deviation (RMSD), (**B**) Radius of Gyration (Rg), (**C**) Root Mean Square Fluctuation (RMSF), (**D**) Solvent accessible Surface Area (SASA).

The dynamic behavior and stability of the complex interactions between the vaccines and their receptors have been revealed by implementing 100 ns MDs at 300 °K. The vaccine’s structural adjustment and stability in the binding domains of the selected receptor were assessed using statistical measures, including root mean square deviation (RMSD), radius of gyration (Rg), root mean square fluctuation (RMSF), solvent accessible surface area (SASA) (Fig. 6A–D) and hydrogen bond analysis. Effective information on the propensity of protein structures to enlarge during MDs is provided by the Rg. During the simulation, it was found that the Rg of the TLR3-DENV complex remained constant, averaging 3.75 nm (Fig. 6B). The protein is less compact and has less folded stability, the greater the Rg. Interestingly, the system’s Rg profile agrees with the complex fluctuation of RMSD and RMSF profiles. The root mean square deviation (RMSD) was used to assess the simulation’s stability. The structural changes that occurred during the MD are demonstrated by the RMSD values^24^. Based on the movement of the RMSD curve within the 2 nm range, the RMSD plots for all proteins showed that each system stabilized immediately and remained stable for the entire period of the simulation (Fig. 6A). According to these graphs, every system appeared to be stable enough for further study. To understand the flexibility of specific amino acids during the simulation, the root-mean-square fluctuation (RMSF) analysis was conducted. Compared to other amino acid residues in the complex, those involved in significant interactions are often more flexible and less restricted. Strong hydrogen bonding between TLR3 has resulted in reduced variations (wavy pattern) as can be shown from the RMSF plot (Fig. 6C). The other component of DENV is fluctuating more. When the two plots are compared, it can be seen that the complex, including TLR3, is exhibiting somewhat fewer shifts, which suggests improved binding between the interaction partners and a stronger complex.

Further, the surface area around the protein for binding to the DENV vaccine was calculated using solvent-accessible surface area (SASA) analysis. A protein surface area that is adequately exposed to interact with nearby solvent molecules is known as the solvent-accessible surface area or SASA. SASA, which is defined by a hypothetical solvent sphere centre with a van der Waals contact with the molecule’s surface, is thought to be a decisive factor for studies on protein stability and folding^25^. The total area of the TLR3-DENV couples complex was about 600 nm2 after 100 ns (Fig. 6D). We started by counting the H-bonds that connected the protein and the DENV vaccine complex. It was found that they varied during the simulation. H-bond occupancy is the percentage of time that an H-bond forms between a ligand atom and any of the protein’s interacting residues. Studying hydrogen bonds can provide insightful information about the stability of a peptide-protein complex and can be applied to change a lead molecule to boost its activity. The angles and distances between the donor, hydrogen, and acceptor atoms can be used to calculate the strength of a hydrogen bond. A larger distance and a smaller angle away from 180^0^ suggest a weaker relationship. Furthermore, it was observed that initially, about 10 H-bonds were formed with an increase in H-bonds during 0–10 ns and a sudden drop in H-bonds. Later, there was an increase at 25 to 40 ns, following stability during the entire 100 ns simulation between the TLR3 and DENV epitope construct.

### Mapping correlated motions and conformational dynamics

**Fig. 7:**
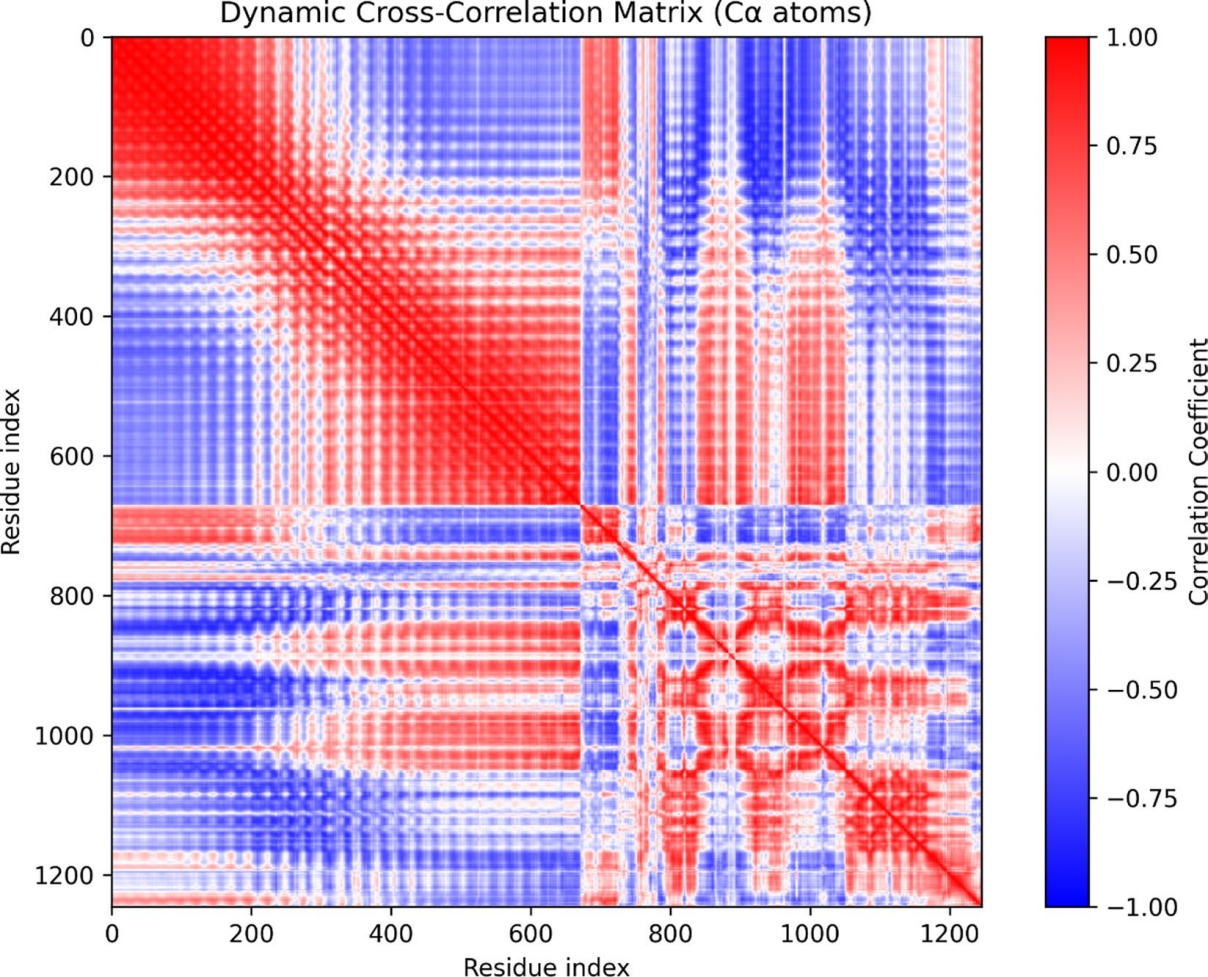
Dynamic cross-correlation matrix reveals cooperative and antagonistic residue motions in DENV Vaccine-TLR complex during simulation.

The DCCM plot represents the correlated and anti-correlated motions of Cα atoms across the DENV-TLR3 protein-vaccine complex during the molecular dynamic’s simulation (Fig. 7), providing rich insight into the mechanistic basis of complex formation and allosteric communication. The matrix reveals significant shifts in residue motion patterns, particularly around residue indices 220–280, 620–700, 800–950, and 1100–1200. In these regions, blocks of positive correlation (red) and negative correlation (blue) are pronounced, signifying substantial changes in the directionality and cooperativity of atomic movements due to complexation or vaccine-induced conformational adaptation. For example, between indices 220 to 280 and 620 to 700, highly anti-correlated motions are observed, likely attributable to the interaction interfaces or domain rearrangements created upon DENV binding to TLR3. Moreover, the regions 800–950 and 1100–1200 illustrate strong intra- and inter-domain communication, as suggested by extensive contiguous blocks of positive correlation that are frequently associated with large-scale cooperative motions and may represent domains stabilizing the DENV-TLR3 binding interface. The boundaries between these correlation clusters imply well-defined hinge points or structural transitions, potentially modulated by the vaccine construct to enhance immunogenicity or optimize receptor activation. These observations are crucial because they underscore the dynamic nature of the complex and highlight specific residue stretches that serve as communication hotspots or flexible segments facilitating allosteric transition. Given that DENV represents a vaccine construct, the conformational changes spotted in the plot not only validate stable complex formation but also reinforce the feasibility of DENV-induced allosteric modulation in TLR3, supporting both efficient binding and downstream signalling relevant for immune recognition. Overall, the DCCM analysis provides a molecular-level understanding of the cooperative and antagonistic motions in the DENV-TLR3 complex, identifies the critical residue ranges, which specifically at indices 220–280, 620–700, 800–950, and 1100–1200, where notable changes occur, and implicates these stretches as key sites for vaccine-driven functional and structural adaptation essential for immunological efficacy.

### Dimensional reduction and dynamic correlation analysis via PCA

**Fig. 8:**
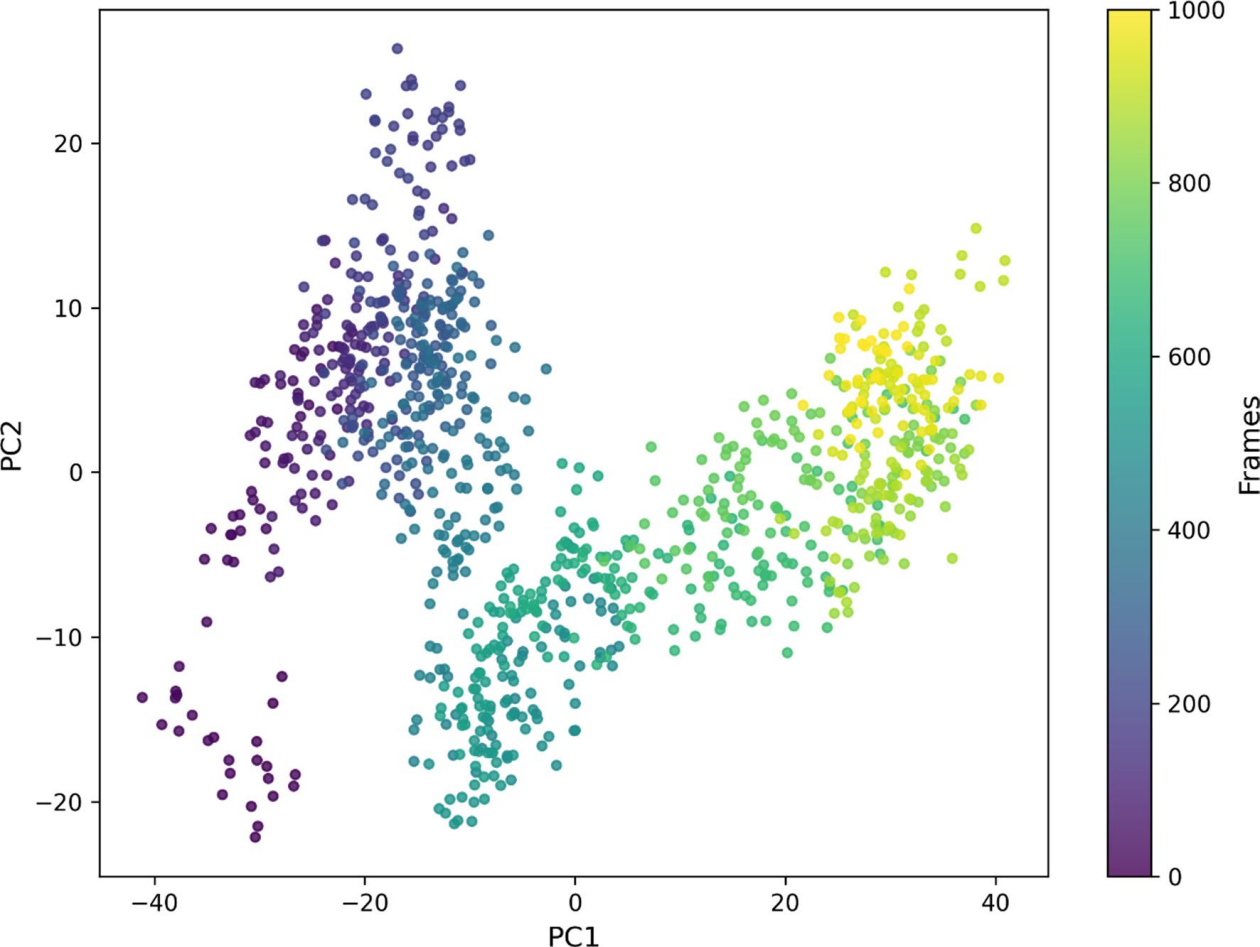
Conformational distribution of TLR3-DENV protein complex captured by principal components (PC1 & PC2) across molecular dynamics frames.

The PCA scatter plot provides a comprehensive visualization of the conformational landscape sampled by the DENV-TLR3 protein-vaccine complex throughout the molecular dynamics simulation, with each point representing a distinct structural state in the principal component space and colored according to trajectory frame progression (Fig. 8). From the distribution of points along PC1 and PC2, it is evident that the system explores two major conformational basins, as seen by the clustering of points around frame numbers 100–300 (dark purple/blue) and 750–1000 (yellow/green), which correspond to early and late stages of the simulation, respectively. Initially, the complex is predominantly confined to a narrow conformational regime around PC1 = −30, PC2 = 10, suggesting a stable association or relatively rigid interface possibly dictated by initial docking orientations. As the simulation advances, there is a marked shift around frames 350 to 600; here, the molecular ensemble transitions to a broader span of conformational space along both PC1 (shifting toward positive values) and PC2 (scattering between − 15 and 10), implying substantial structural rearrangements, potentially induced by vaccine construct-mediated interactions with TLR3 or by the accommodation of domain movements crucial for signalling. By frame 600 and beyond, the system stabilizes in a distinct subspace (PC1 ≈ 35, PC2 ≈ 5–15), coinciding with the intensification of yellow/green points, indicating that the complex has reached a functionally relevant state characterized by increased global flexibility and possibly optimized antigenicity or receptor activation. Such a trajectory illustrates a dynamic adaptation process, wherein the binding of the DENV vaccine construct to TLR3 first restricts, then progressively diversifies, and eventually stabilizes the conformational repertoire of the complex. These findings point to the presence of critical conformational shifts primarily between frame indices 350 and 600, marking the region where major transitions and structural evolution occur in response to molecular interaction and allosteric communication. Overall, the PCA analysis underscores the non-static nature of the DENV-TLR3 complex during MD simulation and corroborates the functional importance of conformational sampling, especially at transition frames, which may relate to potent immunological outcomes or vaccine efficacy.

### Thermodynamic profiling and free energy calculations of vaccine-TLR3 interactions via MM/PBSA

**Fig. 9:**
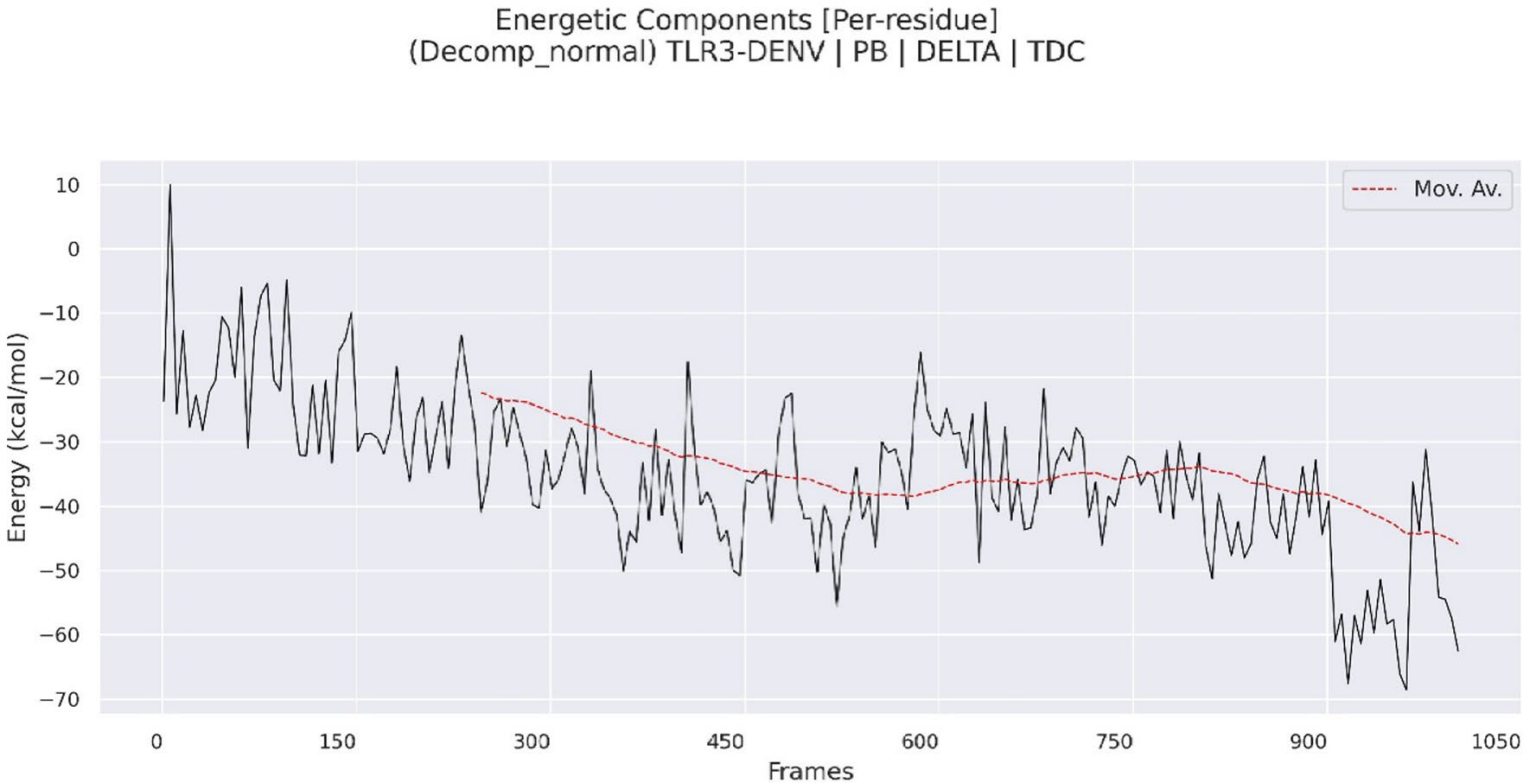
Temporal evolution of binding free energy: MM/PBSA profile of TLR3-DENV complex stability across MD simulation frames.

The total MM/PBSA energy profile for the DENV-TLR3 protein-vaccine complex, as shown in the plot complex (Fig. 9), tracks the binding free energy over 1001 molecular dynamics frames and reveals critical insights into the stability and binding affinity of the complex throughout the simulation. Initially, the binding free energy exhibits higher fluctuations, with values ranging from approximately − 50 to −70 kcal/mol in the first 150 frames, indicative of the system undergoing conformational equilibration as the DENV-TLR3 interface adjusts and stabilizes after complex formation. Significant energy drops-translating to improved binding affinity and stabilization—are observed from frame 300 onward, with a notable stabilization phase: the moving average plateaus and shows reduced variance between frames 300 to 800, fluctuating primarily between − 85 and − 105 kcal/mol. This steady state signals the attainment of a stable binding mode between DENV and TLR3, suggesting that the vaccine construct secures a robust interface with the immune receptor during this period. After frame 900, the binding free energy further decreases, episodically reaching values as low as −135 kcal/mol, which could indicate either enhanced interaction, possibly owing to cooperative domain rearrangement or the formation of additional stable contacts, or reflect local conformational sampling of particularly favourable binding modes. The red dashed moving average confirms this trend by smoothing the energy landscape and highlighting the long-term stabilization and moderate improvement in binding affinity. These dynamics emphasize the importance of excluding early, highly fluctuating frames when calculating representative MM/PBSA values, as well as the necessity of thorough sampling to capture transient but meaningful low-energy events near the end of the trajectory. Overall, the MM/PBSA analysis demonstrates that the DENV construct, complex with TLR3 reaches a relatively stable interaction pattern over time, with noticeable energetic transitions during early (0–150 frames) and late (after ~ 900 frames) simulation windows.

**Fig. 10:**
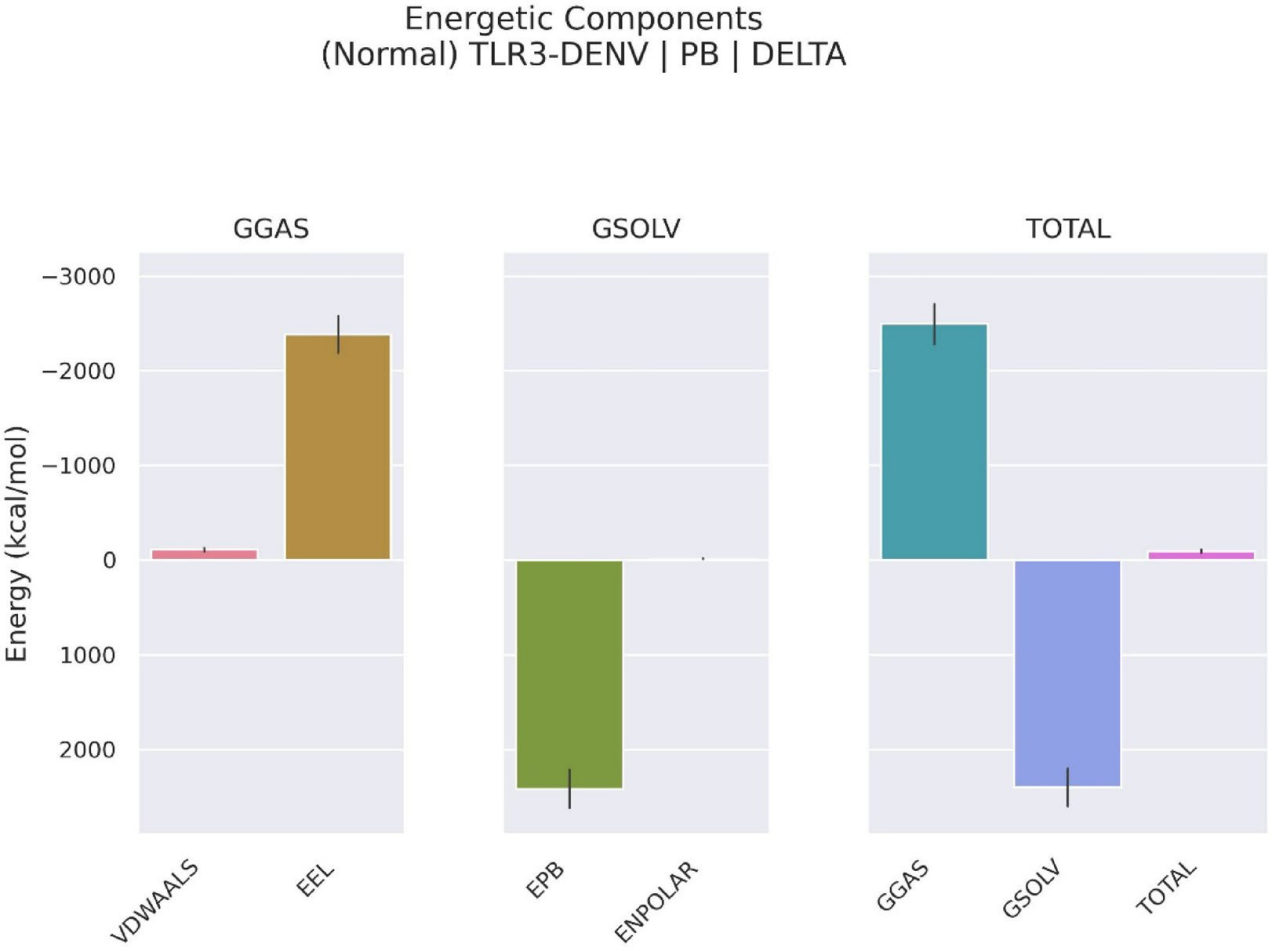
Energetic component analysis of DENV vaccine construct interaction with TLR: gas-phase, solvation, and total binding contributions.

The MM/PBSA summary (Table 3) and energetic component analysis (Fig. 10) for the DENV-TLR3 protein-vaccine complex reveal that the interaction is highly stable and strongly favourable for binding. The total binding free energy averages around − 92.6 kcal/mol, suggesting a potentially favourable interaction across the sampled conformational frames. This stability is principally driven by the electrostatic (EEL) contribution, which registers a highly negative value (−2383.8 kcal/mol) and is supported by van der Waals (VDWAALS) interactions (−109.4 kcal/mol), together comprising the gas-phase total (GGAS) at −2493.2 kcal/mol. These results highlight that the formation of the TLR3-DENV complex is energetically dominated by charge–charge attractions at the interface, leading to strong molecular recognition essential for immune function and vaccine efficacy. However, solvation energy (GSOLV) exerts a substantial counteracting effect, driven by a large positive polar term (EPB, + 2415.5 kcal/mol), which reflects the energetic penalty of desolvating charged and polar groups when moving from solvent exposure into the protein-protein interface. The non-polar solvation component (ENPOLAR, −14.9 kcal/mol) makes only a minor stabilizing contribution. Importantly, the overall total energy (GGAS + GSOLV) remains strongly negative, reaffirming the net gain in binding affinity despite solvation penalties, and supporting the premise that tight and specific binding is achieved in this construct. Error analysis across simulation frames shows low variance, enhancing confidence in the results’ consistency. Taken together, these findings provide insight into the molecular forces that may underpin vaccine–receptor interactions, largely influenced by electrostatic contributions. While these computational results suggest possible receptor engagement, experimental studies are required to determine any functional immunomodulatory effects. This energetically detailed view decisively highlights the charge-based molecular architecture and the nuanced balance between intermolecular stabilization and solvent exclusion penalties that govern the fidelity and specificity of DENV-mediated TLR3 binding.

**Table 3:** Summary of binding free energy components calculated by MM/PBSA for the TLR3–DENV vaccine complex over selected MD trajectory frames.

Sl. no	Energy Component	Average
1	VDWAALS	−109.371 $\:\pm\:$ 15.637
2	EEL	−2383.83 $\:\pm\:$ 111.151
3	EPB	2415.512 $\:\pm\:$ 91.850
4	ENPOLAR	−14.8701 $\:\pm\:$ 0.987
5	GGAS	−2493.2 $\:\pm\:$ 113.009
6	GSOLV	2400.642 $\:\pm\:$ 91.856
7	TOTAL	−92.5541 $\:\pm\:$ 145.632

*VDWAALS: van der Waals; EEL: electrostatic; EPB: positive polar term; ENPOLAR: non-polar solvation component; GGAS: gas-phase total; GSOLV: solvation energy.

**Fig. 11:**
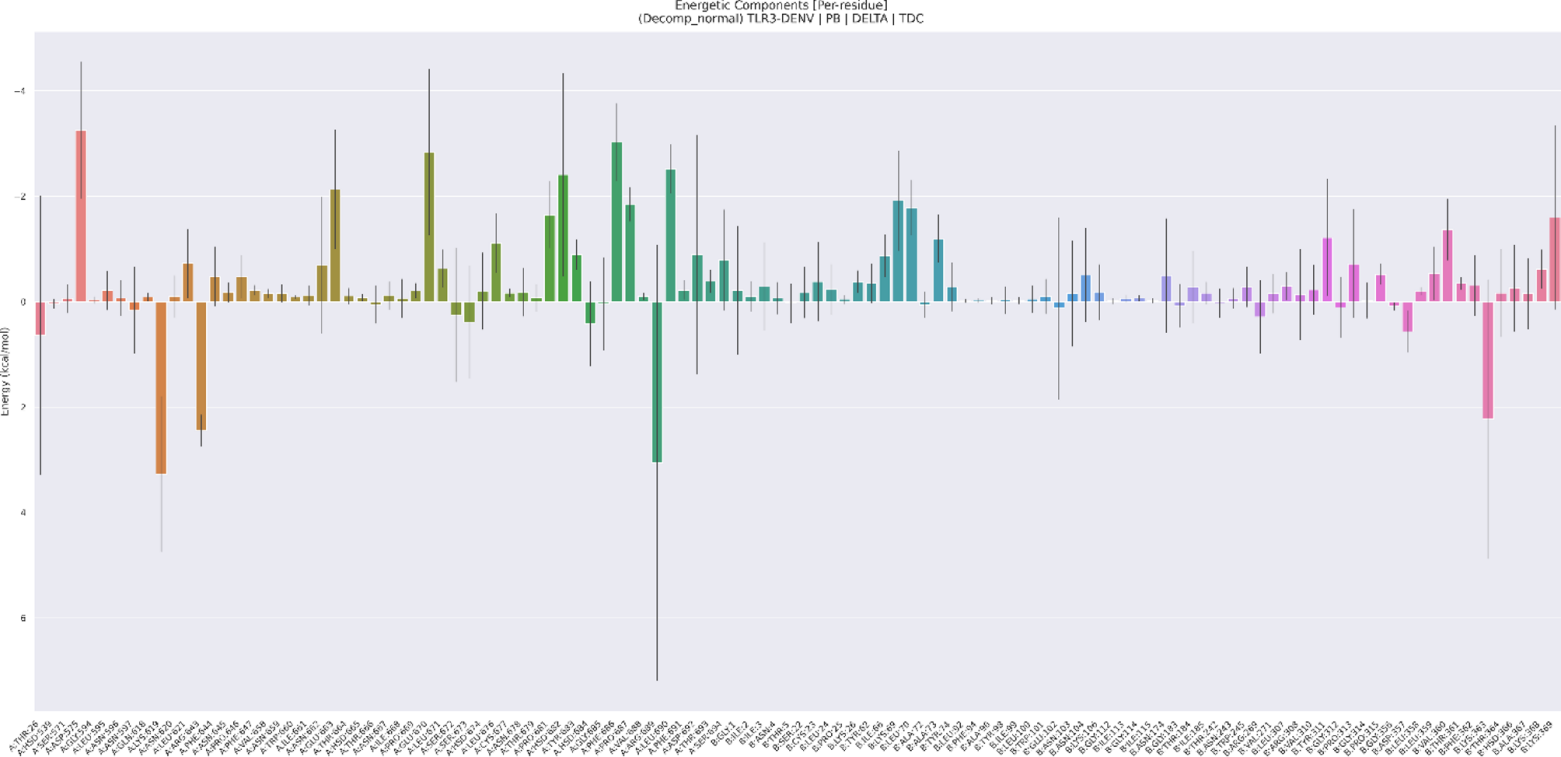
Mapping energetic hotspots: Per-residue decomposition reveals binding determinants at the DENV-TLR protein–protein interface.

The per-residue energy decomposition plot from the MM/PBSA analysis of the DENV-TLR3 protein-vaccine complex provides a detailed snapshot of the energetics that underpin complex formation and stability (Fig. 11). This bar graph illustrates the participating residues and their respective contributions to binding free energy, with notable fluctuations and outliers pointing to hotspots of interaction or instability. Classically, residues on the TLR3 side (indices with A_ prefix, spanning 1–671) and those on the DENV construct (B_ prefix, 672–1246) exhibit substantial energetic diversity, but the most pronounced changes in energy occur at specific points such as: A_Trp36, A_Gln64, A_Asp85, A_Glu86, A_Ser89, A_Lys110, and A_Asn667. These residues show strong negative energy values (around − 2 to −4 kcal/mol), indicative of energetically favourable interactions critical for binding affinity and interface stabilization. Notably, DENV construct residues including B_Ser20, B_Tyr44, B_Leu74, and B_Gln156 display significant energy variation, reflecting their direct involvement in the protein-protein interface. Positive energy spikes, for example, at A_MSE61 and A_Lys626, reveal either unfavourable contacts or local steric/electrostatic clashes, which may modulate transient binding and dynamic rearrangement during complex formation. The diversity of contributions across the sequence landscape emphasizes the multifaceted nature of protein-protein recognition, where several distributed clusters cooperatively drive specificity and thermodynamic stability. Error bars show the standard deviation across simulation frames, suggesting regions of greater conformational flexibility and possible roles in allosteric communication. Importantly, high-magnitude energetic components cluster around regions 60–110, 620–670 (TLR3) and 10–70, 140–160 (DENV), identifying these stretches as key determinants in the stabilized complex and potential targets for mutational or functional analysis.

### Immune simulation of vaccine candidate

**Fig. 12:**
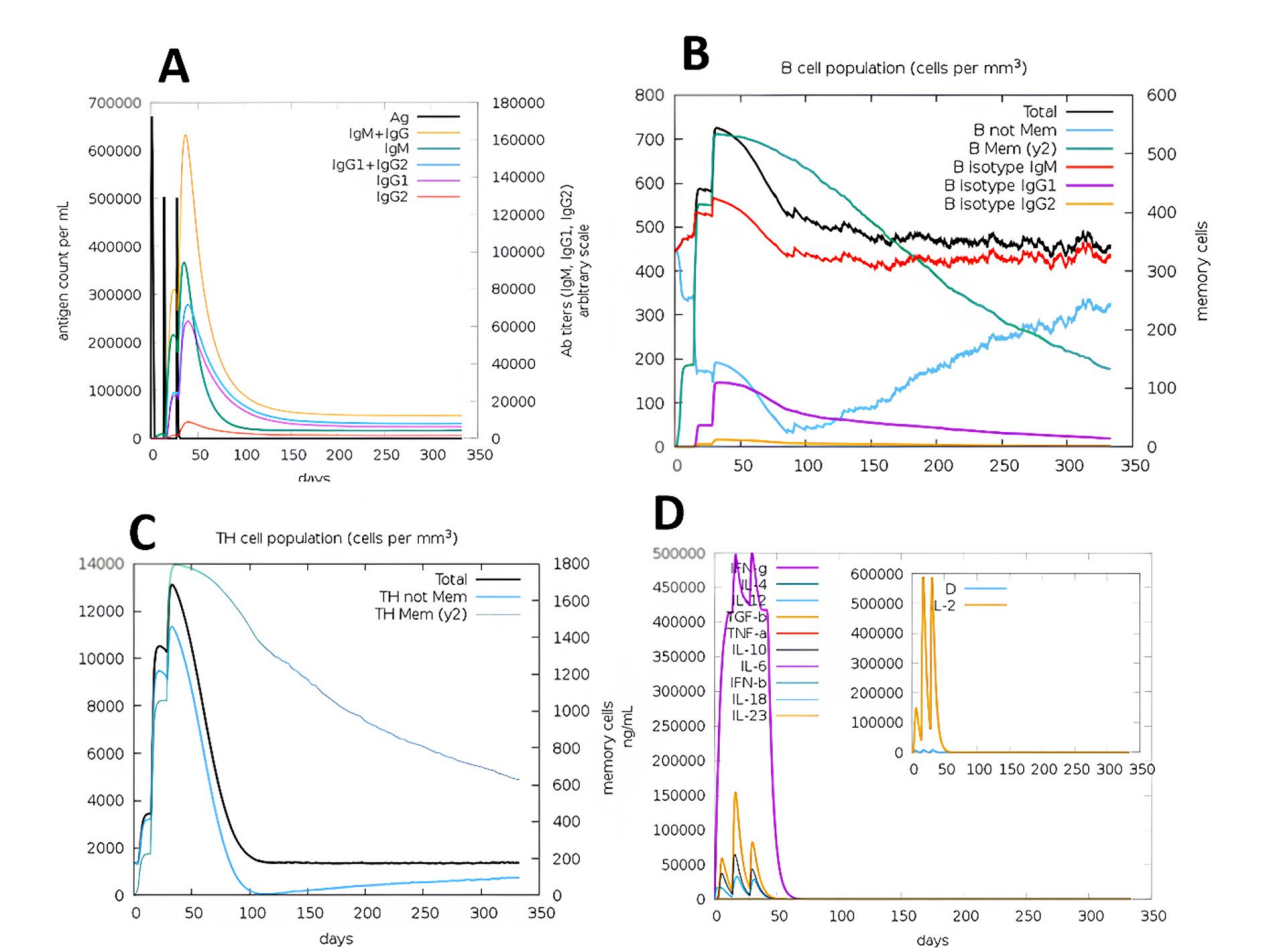
Immune simulation of DENV-1 & −3 vaccine constructed by IMMSIM server (**A**) Immunoglobulin synthesis in response to antigen encounter. (**B**) Population of In silico B Lymphocytes, it represents scale of memory B cells. (**C**) Number of T lymphocytes, it shows the count of memory T cells. (**D**) The extent of the concentration of cytokines and interleukin.

The developed DENV-1 & DENV-3 multi-epitope vaccine construct was shown to be able to elicit a strong and long-lasting immune response in an in silico immunological simulation conducted using the C-IMMSIM server. To imitate primary and secondary immunological exposures, a three-dose vaccination regimen was created (Fig. 12). With a noticeable rise in IgG1 + IgG2 levels after booster doses, the secondary and tertiary reactions were noticeably stronger than the main one, suggesting successful isotype switching and the development of immunological memory. The formation of long-term humoral immunity was confirmed by the initial reaction, which was marked by a brief increase in IgM, which was followed by raised IgG titers following antigen reduction. Multiple B cell isotypes persisted throughout the simulation period, indicating memory B cell production and continuous isotype class change. Significant increases in helper T-lymphocytes (HTLs) and cytotoxic T-lymphocytes (CTLs) revealed that the multi-epitope vaccination design concurrently produced a strong cell-mediated immune response. Durable adaptive immunity was demonstrated by the persistence of memory CTL and HTL populations across time. Dendritic cell numbers stayed constant, allowing for ongoing antigen presentation, whereas macrophage activity gradually increased with each dose, improving antigen clearance among innate immune components. Furthermore, it was hypothesized that a balanced cytokine milieu favouring Th1-dominated antiviral responses with regulated immune regulation was activated by higher levels of IFN-β and TGF-β (Supplementary Figures S2 & S3). The simulation outputs suggest the possibility of coordinated humoral and cellular responses and indicate theoretical immunogenic potential; however, experimental studies are needed to confirm these effects.

### In silico vaccine cloning

**Fig. 13:**
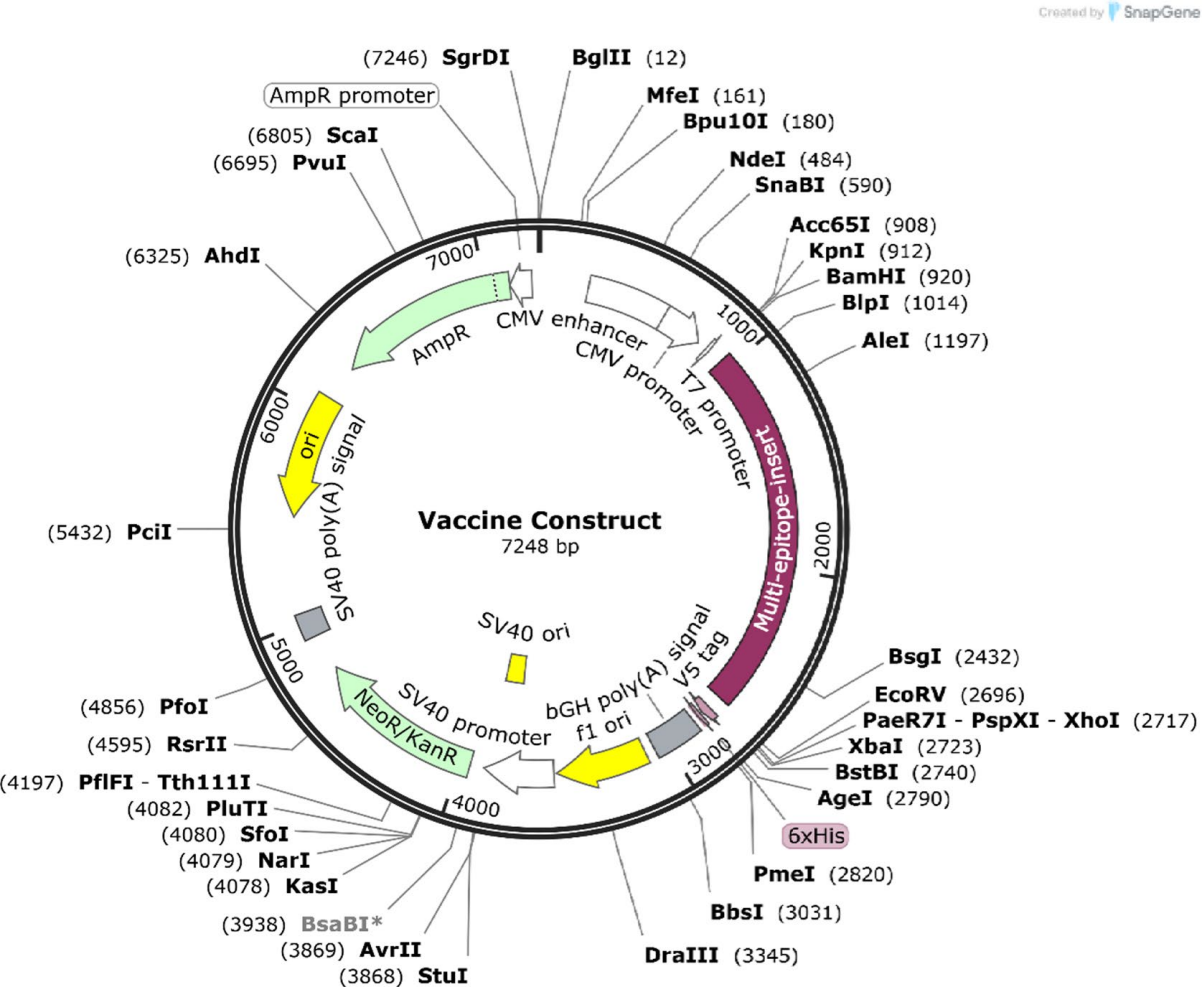
In silico cloning of multi-epitope vaccine sequence (red) cloned in pcDNA3.1/V5/His-TOPO/LacZ vector.

Jcat server codon optimized in silico vaccine candidate for *E. coli* protein expression. The vaccine has 1725 nucleotides and 68.75% GC. SnapGene software was used to clone the design into pcDNA™3.1/V5-His-TOPO^®^ (Fig. 13).

## Discussion

In recent years, dengue fever, which can range in severity from moderate to severe, has spread more widely and been connected to higher death rates. Despite being common, there is presently no safe vaccination for the condition, and it is only treated symptomatically^26^. Despite having licenses, the effectiveness of two vaccines (Dengvaxia and Qdenga) against seronegative individuals varied depending on the dengue serotype. Many vaccines are still in the development stage and are now being tested in clinical settings^22,27^. Compared to the in silico process, the conventional approach to vaccine development is typically more expensive, time-consuming, and prone to failure^28^. Based on the requirements of the immune response, an immunoinformatic approach can be used to build a vaccine at the humoral or cellular level. The vaccine will be designed using the antigenic regions of the pathogenic genome that can trigger an immune response, and its physicochemical properties will be assessed^29,30^. Due to their specificity, low production costs, reduced allergenicity, and aversion to unwanted immune responses, epitope-based vaccinations provide distinct advantages over traditional ones^31,32^. Compared to single-epitope and conventional vaccinations, a multi-epitope vaccine offers unique properties. Because TCRs from different cell subsets can recognize several MHC I and MHC II epitopes, in silico designed multi-epitope vaccines can avoid issues related to in vitro antigen production and the difficulty of culturing the pathogens. Additionally, humoral and cellular immune responses can be triggered simultaneously by the overlapping HTL, CTL, and B cell epitopes. Phase I clinical trials have been started for vaccines made with several epitopes, a new area that has already gained notoriety. In vivo efficacy with protective immunity has been proven by these vaccines^33,34^.

Compared to the other two serotypes, DENV-1 and DENV-3 seem to cause more severe illness in primary infections. Dengue co-infection outbreaks of DENV-1 and DENV-3 have also been documented, despite the fact that many dengue infections are linked to a single serotype^35^. We used E and NS1 as antigens from DENV-1 and DENV-3 in our investigation to create a multiepitope-based vaccine. The proteins E and NS1 were recovered and put through antigen prediction. Antigenic epitope prediction is useful for studying the body’s defence system and for creating vaccine components. Certain areas of a protein’s surface that B cell antibodies uniquely recognize are known as antigenic epitopes^36^. Percentile rank was used to predict how peptides would attach to MHC I and MHC II. The percentile rank was chosen up to 0.5 for MHC I and up to 2.5 for MHC II. A lower percentile rank indicates a higher binding capacity. The CTL epitopes are in charge of creating enduring immunity that can destroy virus-infected cells and circulating viruses. On the other hand, HTL epitopes are associated with humoral and cellular immune responses, triggering the development of protective CD8 + T cell memory and activating B cells through the induction of a CD4 + helper response^33,37^. A vaccine was designed using specific peptides following the prediction of antigenicity, IFN γ score, and B cell epitope. Using human beta-defensin as an adjuvant, the multi-epitope vaccine was created using the chosen epitopes of MHC I, MHC II, and B cells. Adjuvants will boost the vaccine’s antigenicity, while peptide-based vaccines will have poor immunogenicity^38^. Epithelial cells contain beta-defensin, a low molecular weight antimicrobial peptide^39^. Linkers are used to join all of the peptides that contain an adjuvant. The flexibility, stiffness, and cleavability of proteasomal systems are preserved in the vaccination thanks to these linkers^40^. The EAAAK linker was utilized to join the adjuvant with epitopes and improve protein stability. Linkers are little amino acids. The ubiquitous spacer GPGPG linker aids in the induction of T helper cell responses. Additionally, it is essential for preventing junctional immunogenicity and promoting the recovery of individual epitope immunogenicity. By inhibiting the generation of antibodies against the peptide sequence that happens when individual epitopes are joined linearly, AAY and KK linkers aid in immunogenicity and decrease junctional immunogenicity^41,42^.

Understanding the protein’s physicochemical characteristics and how it folds into its secondary structure is necessary for vaccine creation. The vaccine’s estimated molecular weight is 62.63 kDa. It was determined to be non-allergenic and antigenic. The vaccination was alkaline as the theoretical isoelectric point was 9.77. With an instability rating of less than 40 (30.63), the protein was stable. The vaccine is thermostable according to its aliphatic index, and a negative GRAVY score means that the vaccine and water molecules interact better, which increases protein solubility^43,44^. The interaction between the vaccination and the host cell was examined after the physicochemical characteristics were assessed.

Human Toll-like receptors (TLR) are type-1 transmembrane proteins found on skin epithelial cells, in the gastrointestinal, urogenital, and respiratory tracts, as well as in innate immune cells. When TLRs come into contact with any foreign objects, they trigger the cascade system, which activates the host immune system and then adaptive immunity. Targeting TLR3 in a multi-epitopic dengue vaccination is a safer and more effective approach because it recognizes viral double-stranded RNA and generates type I interferons and pro-inflammatory cytokines that initiate an antiviral immune response. Better antigen presentation, enhanced dendritic cell maturation, and the generation of neutralizing antibodies are the outcomes of this. Targeting TLR3 is theoretically considered a promising strategy for enhancing antiviral signaling, although its impact on immunopathology or ADE cannot be determined without experimental evidence^45,46^. Through the use of molecular dynamics simulation, the relationship between vaccination and TLR 3 receptors was investigated. The protein has poorer folding stability and compactness, according to the Rg plot. Both TLR3 receptors’ structural changes throughout the MD were sufficiently stable, according to the RMSD plot, and the TLR3 receptor’s complex binding was enhanced, according to the RMSF analysis. The DENV-TLR3 protein vaccine combination showed good stability and positive binding, according to an MM/PBSA study. An average binding free energy of −92.6 kcal/mol demonstrated a strong connection across multiple conformational frames.

The immune simulation predicted the potential for both innate and adaptive immune activation, although these outcomes require experimental validation. The vaccine has a GC content of 68.75% and was codon optimized in the *E. coli* system. It was subsequently cloned to the vector of mammalian expression. Increased IFN-γ levels during immunological research suggest that the vaccine’s design can produce an antiviral effect. By preventing viral replication, IFN-γ is essential for cell-mediated immunity^47^. The results imply that vaccinations can interact with immunological receptors in an efficient manner.

When our present study was compared with a recent in silico designed dengue vaccine, it showed both advancement and alignment. One study revealed the immunogenic potential of the DENV-3 vaccine construct^48^, and another study demonstrated a stable interaction between DENV-2 and the TLR4 receptor^49^. At the same time, both studies targeted a single serotype, in contrast to another study where an NS5-based vaccine was developed for all serotypes but lacked the energetic or structural stability of the construct^50^. In comparison to past investigations, the current work on DENV-1 and DENV-3 confirms conformational stability and interaction energetics during co-infection instances, which have rarely been addressed in previous research. These findings highlight the computationally derived features of the construct and suggest its potential relevance for further investigation in the context of DENV-1 and DENV-3 co-infection.

## Limitations

This work is limited to computational predictions, despite incorporating several immunoinformatics and structural bioinformatics techniques. The study focuses only on the E and NS1 antigens of DENV-1 and DENV-3, which may not fully represent the variation seen in natural infections. While the predictions of T cell and B cell epitopes, antigenicity, allergenicity, and physicochemical properties offer valuable preliminary insights, they cannot replace in vitro and in vivo validation. A variety of algorithms were employed for predicting B cell and T cell epitopes, structural modelling, and immune simulations. However, these tools are based on idealized assumptions and cannot fully replicate human immune responses, HLA variability, cross-reactivity, and antibody-dependent enhancement. The study of ligand-receptor interaction and its stability can benefit from the results of molecular docking, molecular dynamics simulations, PCA, DCCM, and MM/PBSA calculations; however, these are all computational measurements that may differ from the underlying biological interactions. Codon optimization and in silico cloning do not guarantee the structural stability or expression of the vaccine construct in a biological system; therefore, experimental validation is necessary. Overall, the study supports the potential of the designed multi-epitope vaccine, but further experimental validation is needed.

## Conclusion

This study presents a comprehensive immunoinformatics-based approach to design a multi-epitope vaccine targeting co-infection by DENV-1 and DENV-3, using epitopes derived from the NS1 and E proteins. Computational analysis predicted the construct to be antigenic, potentially immunogenic, and non-allergenic. Structural evaluations suggested stability, and docking predicted a favourable interaction with TLR3; however, these findings require validation through laboratory experiments. The vaccine also showed potential to elicit robust B cell, T cell, and cytokine-mediated responses, indicating its ability to activate both innate and adaptive immune pathways. Furthermore, codon optimization results support its suitability for expression in mammalian systems. To the best of our knowledge, this is the first study to propose a multi-epitope vaccine specifically targeting DENV-1 and DENV-3 co-infection using immunoinformatics pipeline. While these findings offer promising insights, further in vitro and in vivo studies are essential to validate the vaccine’s efficacy and safety. The data generated here lays a strong foundation for future experimental investigations and may contribute to the broader efforts in dengue vaccine development.

## Materials and methods

### NS1 and E protein sequence search for antigen prediction

DENV-1’s (NP_722461.1 and NP_722460.2) and DENV-3’s (YP_001531169.2 and YP_001531168.2) non-structural protein 1 (NS1) and envelope protein (E) amino acid sequences were obtained in FASTA format from the National Centre for Biotechnology Information (NCBI) database (https://www.ncbi.nlm.nih.gov/). Antigen prediction was applied to the protein sequences that were obtained from NCBI^31^.

### Predicting T and B cell epitopes

For the NS1 and E protein of DENV-1 and − 3, the IEDB server (Immune Epitope Database and Analysis Resources) (http://www.iedb.org/) was used to predict the 9-mer cytotoxic T lymphocyte (CTL) and 15-mer helper T lymphocyte (HTL) epitopes. Percentile rank was used to select the potential MHC class I and II binding peptide^51^. In order to assess the immunogenic potential of MHCI predcited epitopes identified through the IEDB server were employed to IEDB MHC I Immunogenicity tool for their likelihood of interaction with T-cell receptors. Using in silico analysis to identify the B cell epitope in the antigen is essential for vaccine design. The ABCpred server (https://webs.iiitd.edu.in/raghava/abcpred/ABC_submission.html) was used to predicted the potenital B cell epitope on default parameter ^52^. The linear epitope region that aids in the selection of the synthetic vaccine will be identified with this server. ABCpred server predicts B cell epitopes - with 65.93% accuracy using a partly recurrent neural network^53^. 

### Interferon-γ (IFN-γ) epitope prediction

It has been demonstrated that IFN-γ influences both innate and adaptive immunity, greatly bolstering host defences. IFN signalling affects the entire antigen processing and presentation process. Natural killer cells (NK) and natural killer T cells (NKT) are the main cells of the innate response that produce IFN. IFN-γ is essential for Th1 immune responses and regulates T cell activity at every stage. IFN-γ is a cytokine that activates CD4 + T lymphocytes by causing MHC class II expression^54,55^. In order to create the most effective subunit vaccines, the IFN epitope server (http://crdd.osdd.net/raghava/ifnepitope/design.php) was utilized to identify the epitopes that can trigger IFN-γ from MHC II binding epitopes. IFN-gamma versus non-IFN-gamma was the selection model, and motif and SVM hybrid methods were used to predict IFN-γ^56^.

### Antigenicity and allergenicity determination

Antigenicity, is the term used to describe the binding of an antigenic epitope to the receptors of B cells or T cells. The capacity of an antigen to elicit an immunological response and produce antibodies is known as immunogenicity^57^. The VaxiJen v2.0 (http://www.ddg-pharmfac.net/vaxijen/VaxiJen/VaxiJen.html) was used to determine the likely antigen and with - prediction accuracy ranges from 70 to 89%. The antigenicity of epitopes was predicted using the virus as the target organism, a threshold value of 0.4 . To avoid host cross-reactions, the allergenicity of the vaccination must be ascertained. The prediction tool utilized is AllerTOP v2.0 (https://www.ddg-pharmfac.net/AllerTOP/)^51,58^.

### Construction of a multi-epitope vaccine

Several immunoinformatic techniques were used to analyze the peptides that were predcited as potenital T & B cell epitopes. The selected epitopes underwent additional processing in order to create the vaccine candidate. The vaccine’s N terminal had a 45 amino acid beta-defensin adjuvant, which is one of the adjuvants that helps strengthen the immune response. The linker that was employed next to the adjuvant close to the MHC I epitopes, was EAAAK. Linkers like AAY, GPGPG, and KK were employed in the development of the vaccine candidate between MHC I, MHC II, and B cell epitopes, respectively. In order to enhance antigen processing and presentation and preserve the independent immunogenic actions, linkers were employed^31^.

### Physicochemical property prediction

An online program named ExPASy-ProtParam (http://web.expasy.org/protparam/) was used to analyze the physical and chemical characteristics of the NS1 and E proteins of DENV-1 and DENV-3 as well as their developed vaccine based on pK values. The number of amino acids, theoretical pI, molecular weight, amino acid composition, total number of positively and negatively charged residues, atomic composition, extinction coefficient, half-life, instability index, aliphatic index, and grand average of hydropathicity (GRAVY) are among the physical and chemical characteristics described in this server^59^.

### Prediction of secondary and tertiary structure and validation and refinement of 3D structure

The secondary structure of the NS1 and E proteins was predicted using the web server PSIPRED (http://bioinf.cs.ucl.ac.uk/psipred), and the primary amino acid sequence was used to build the vaccine. The output from PSI-BLAST (Position-Specific Iterated BLAST) is analyzed by PSIPRED, which incorporates two feed-forward neural networks^60^. The tertiary structure of the construct was predicted using the sequence-to-structure-to-function method of the I-TASSER service^61^. In order to find any possible mistakes, the built entity’s 3D structure must be validated. For validation, the Ramachandran plot was created using PDBsum (http://www.ebi.ac.uk/thornton-srv/databases/pdbsum/Generate.html), a visual database that builds the vaccine’s tertiary structure. A PROSA analysis was conducted for additional validation of protein structure. This led to the computation of a comprehensive quality score for the given structure. If this score deviates from a defined range for native proteins, the structure is likely erroneous.

### Disulfide engineering of the vaccine construct

Disulfide engineering is a powerful computational approach designed to enhance the structural stability and stiffness of proteins by including new disulfide bonds through targeted cysteine substitutions at particular residue locations. This method reinforces the protein’s structural integrity, hence improving its resistance to denaturation and destruction. This study employed the Disulfide by Design 2 (DbD2) server to systematically identify residue pairings in the modelled vaccine design that demonstrate suitable geometric and energy characteristics for possible cysteine mutation and disulfide bond formation. The found residue pairs were examined for their potential in disulfide engineering to enhance the structural integrity of the developed vaccination candidate [Craig DB, PMID: 24289175].

### Simulation of molecular dynamics (MD) and molecular Docking

During viral infection, Toll-like receptors (TLRs) trigger the innate immune response by detecting viral elements, notably the envelope proteins. In order to anticipate the mode of interaction between vaccine constructions and TLR3 (PDB ID: 2A0Z), the ClusPro 2.0 server (https://cluspro.org) was used with the default settings. One technique for examining the motion of molecules of interest is MD simulation. The four complexes of multi-epitope vaccines and TLR3 (receptors) that were determined to have the lowest binding energies through docking were subjected to 100ns MD simulations using the GROMACS 2020.6 program^62^. Following their placement in an explicit water box of size 10 with periodic boundary conditions (PBC) and a single-point charge (SPC) water model TIP3P, each complex was solvated separately. The CHARMM36 Additive Force Field was used to add the ligand, Na+, and Cl- ions to the protein in order to neutralize its charge^63^. A 100ns production run was performed on the system after 2000 energy-minimization steps. Using the steepest descent method, energy minimization was achieved with 50,000 steps and a tolerance for 1000 kJ/mol/nm convergence. The system was equilibrated for 150 ps using a standard ensemble of NVT (constant number of particles, volume, and temperature) and NPT (constant number of particles, pressure, and temperature). Particle-mesh Ewald electrostatics summing was used to resolve long-range electrostatic interactions with an order of 4.0 and a Fourier spacing of 0.16 nm. To solve motion equations on box vectors, an extended coupling ensemble of Parrinello-Rahman was employed. The Root Mean Square Deviation (RMSD) approach, which computes the deviations (nm) in the protein trajectory with respect to its backbone, has been used to assess the stability of the multi-epitope-bound TLR3 complex. Lower average root means square fluctuation (RMSF) values indicated more stability. During the MD simulation, the RMSF was used to calculate the average positional dynamicity of each atom in the protein. The Radius of Gyration (Rg) study shows how compact the simulated apo TLR3 structures and multi-epitope bound TLR3 complexes are by measuring the weighted RMS distances of the protein backbone atoms represented by Rg (nm) versus the full simulation timeline^64^.

### Dynamic cross-correlation matrix

The residue dynamic cross-correlation matrix (DCCM) analysis was performed to investigate correlated motions between residues in the DENV-TLR3 protein-vaccine complex system using molecular dynamics (MD) simulation trajectories. The initial trajectory data, consisting of the production run’s coordinates and topology files (step5_production_200ns.xtc and step5_production_200ns.tpr), were loaded into the Python MDAnalysis package, which facilitates trajectory parsing and atom selection^65^. Alpha carbon (Cα) atoms were selected to represent each residue’s position, enabling a residue-level correlation analysis that reduces noise from side-chain flexibility. Before analysis, the trajectory frames were aligned using the protein backbone to remove global translational and rotational motions, ensuring that only internal fluctuations contributed to the correlation calculations^66^. The coordinates of Cα atoms were extracted for all 1001 MD frames, creating a 3D NumPy array. The mean position of each residue over the entire trajectory was computed and subtracted from instantaneous positions to calculate residue positional fluctuations. These fluctuations were then used to compute the correlation coefficients between every pair of residues. The correlation coefficient C ij quantifies the linear relationship between the motions of residues i and j, where values range from − 1 (fully anti-correlated) to + 1 (fully correlated). The resulting DCCM matrix is a symmetric square matrix whose elements indicate the strength and direction of correlated motions between residue pairs across the simulation time. This matrix was visualized as a heatmap, with a blue-to-red color gradient representing anti-correlated to correlated movements. Such analysis highlights regions of the protein that move cooperatively or in opposition, providing insights into allosteric networks, domain motions, and functional dynamics^67^.

### Principle component analysis (PCA)

The PCA analysis was conducted by first extracting the principal components from the molecular dynamics trajectory of the DENV-TLR3 protein complex using GROMACS tools and then visualizing the conformational space explored across the trajectory with Python-based plotting. Specifically, after specifying the protein system via user input in the bash script, GROMACS’s covar command was used to compute the covariance matrix and diagonalize it, yielding eigenvalues and eigenvectors required for principal component analysis^68,69^. The key lines, gmx covar and gmx anaeig, project the MD trajectory onto the top two eigenvectors, PC1 and PC2. These projected data were further processed and visualized using the Python script provided, which loads the PC1 and PC2 coordinates, assigns colors to each frame for gradient visualization, and produces a scatter plot highlighting conformational transitions. Using these projections, the conformational states sampled by the complex were visualized to assess large-scale motions, dynamic shifts, and stability features of the system. Such methodology enables effective dimensionality reduction, revealing the essential collective motions and global flexibility of the TLR3-DENV complex, which are key for understanding functional interactions and allosteric communication relevant to vaccine and receptor efficacy.

### Molecular mechanics poisson-boltzmann surface area (MM/PBSA)

Binding free energy calculations for the DENV-TLR3 protein-vaccine complex were performed using the gmx_MMPBSA tool, which implements the established MM-PBSA approach for estimating the energetics of biomolecular interactions from molecular dynamics trajectories. The index file was carefully constructed to select residues 1–671 as the receptor (Protein A - TLR3) and residues 672–1246 as the ligand (DENV-based vaccine construct), following recommended practices for protein-protein decomposition analysis. This grouping allows residue-level energy decomposition to capture intermolecular interface contributions and is justified by the biological roles of the respective protein and vaccine regions. The input file (mmpbsa.in) was set to include the line print_res = within 6, instructing the software to print binding energy components for residues within 6 Å of the interface, ensuring focus on key interacting residues most likely critical for binding specificity, as supported by literature on interface analysis. For frame sampling, 1001 frames of the trajectory were read with a 5-frame interval. This strategy ensures statistically robust coverage of the entire simulation, balancing computational efficiency and meaningful conformational diversity, as recommended in MM-PBSA protocols where averaging across many snapshots improves result reliability. Calculations employed the single-trajectory (ST) approximation, where the receptor, ligand, and complex coordinates are extracted from the same trajectory, reducing sampling artifacts and ensuring consistent conformational representation. Output files provide binding energy statistics and per-residue decomposition, enabling detailed characterization of the molecular determinants of complex formation and stability between the vaccine construct and receptor. This methodological approach aligns with recent applications of gmx_MMPBSA to protein-protein systems, providing rigor and reproducibility to free energy analysis in biomolecular studies^70,71^.

### Multi-epitope vaccination immune simulation

The immune response to the developed vaccine against DENV-1 and -3 was simulated and predicted by the C-ImmSim (the IMMune system SIMulator) server (http://kraken.iac.rm.cnr.it/C-IMMSIM). Using a position-specific scoring matrix (PSSM) and machine learning approaches, the agent-based model C-ImmSim forecasts immunological interactions and immune epitopes. This model illustrates the humoral and cellular-based immune responses to antigens^72^. It mimics three anatomical areas of mammals, primarily the tertiary lymphatic system, bone marrow, and thymus. 1050 was chosen as the simulation’s parameter value. Three injections were administered at 1, 84, and 168 time steps (each time step in real life is equivalent to eight hours) and at intervals of four weeks. An injection at time = 0) is represented by time-step 1^73^.

### Vaccine candidate optimization and in silico cloning

The multi-epitope vaccine candidate’s codons were optimized, and JCAT was used to analyze the reverse translations. A codon adaptation index (CAI) was used to determine the protein’s expression level. Additionally, the percentage of GC content was disclosed by the JCat output [Grote A; 15980527]. “SnapGene software (www.snapgene.com)” was then used to clone the improved sequence into the pcDNA™3.1/V5-His-TOPO^®^ expression vector.

## Supplementary Information

Below is the link to the electronic supplementary material.

Supplementary Material 1

## Data Availability

This article contains original data and is available from the corresponding author on request.
