# Supplementary material for: In silico design of a multi-epitope vaccine targeting DENV-1 and DENV-3
Source: Sci Rep. 2026 Jan 16;16:5308. doi: 10.1038/s41598-026-35678-0 (PMC12881640; doi:10.1038/s41598-026-35678-0)
Supplement: Supplementary file 1 — Supplementary Material 1 [file 41598_2026_35678_MOESM1_ESM.docx]

**Supplementary Table 1.** Amino acid composition of DENV-1 NS1 and E protein through Protparam

| **Amino acid** | **NS1 Protein** | | **E protein** | |
| --- | --- | --- | --- | --- |
|  | **Residues** | **Pecentage** | **Residues** | **Percentage** |
| Ala (A) | 17 | 4.80% | 30 | 6.10% |
| Arg (R) | 15 | 4.30% | 15 | 3.00% |
| Asn (N) | 16 | 4.50% | 13 | 2.60% |
| Asp (D) | 16 | 4.50% | 19 | 3.80% |
| Cys (C) | 12 | 3.40% | 13 | 2.60% |
| Gln (Q) | 10 | 2.80% | 19 | 3.80% |
| Glu (E) | 27 | 7.70% | 29 | 5.90% |
| Gly (G) | 29 | 8.20% | 49 | 9.90% |
| His (H) | 10 | 2.80% | 10 | 2.00% |
| Ile (I) | 26 | 7.40% | 26 | 5.30% |
| Leu (L) | 21 | 6.00% | 45 | 9.10% |
| Lys (K) | 27 | 7.70% | 36 | 7.30% |
| Met (M) | 9 | 2.60% | 14 | 2.80% |
| Phe (F) | 10 | 2.80% | 18 | 3.60% |
| Pro (P) | 15 | 4.30% | 14 | 2.80% |
| Ser (S) | 22 | 6.20% | 30 | 6.10% |
| Thr (T) | 24 | 6.80% | 56 | 11.30% |
| Trp (W) | 16 | 4.50% | 10 | 2.00% |
| Tyr (Y) | 9 | 2.60% | 8 | 1.60% |
| Val (V) | 21 | 6.00% | 41 | 8.30% |
| Pyl (O) | 0 | 0.00% | 0 | 0.00% |
| Sec (U) | 0 | 0.00% | 0 | 0.00% |

**Supplementary Table 2.** Amino acid composition of DENV-3 NS1 and E protein through Protparam

| **Amino acid** | **NS1 Protein** | | **E protein** | |
| --- | --- | --- | --- | --- |
|  | **Residues** | **Pecentage** | **Residues** | **Percentage** |
| Ala (A) | 18 | 5.10% | 33 | 6.70% |
| Arg (R) | 14 | 4.00% | 13 | 2.60% |
| Asn (N) | 17 | 4.80% | 22 | 4.50% |
| Asp (D) | 11 | 3.10% | 15 | 3.00% |
| Cys (C) | 12 | 3.40% | 13 | 2.60% |
| Gln (Q) | 10 | 2.80% | 19 | 3.90% |
| Glu (E) | 28 | 8.00% | 35 | 7.10% |
| Gly (G) | 28 | 8.00% | 52 | 10.50% |
| His (H) | 8 | 2.30% | 11 | 2.20% |
| Ile (I) | 20 | 5.70% | 31 | 6.30% |
| Leu (L) | 28 | 8.00% | 41 | 8.30% |
| Lys (K) | 24 | 6.80% | 35 | 7.10% |
| Met (M) | 9 | 2.60% | 14 | 2.80% |
| Phe (F) | 6 | 1.70% | 16 | 3.20% |
| Pro (P) | 15 | 4.30% | 16 | 3.20% |
| Ser (S) | 23 | 6.50% | 24 | 4.90% |
| Thr (T) | 31 | 8.80% | 46 | 9.30% |
| Trp (W) | 17 | 4.80% | 10 | 2.00% |
| Tyr (Y) | 11 | 3.10% | 10 | 2.00% |
| Val (V) | 22 | 6.20% | 37 | 7.50% |
| Pyl (O) | 0 | 0.00% | 0 | 0.00% |
| Sec (U) | 0 | 0.00% | 0 | 0.00% |

**Supplementary Table 3.** Predicted MHC I epitopes for NS1 protein of DENV-1 & 3 using IEDB server

| **Sl. No.** | **Start** | **End** | | **Peptide** | **Allele** | | **Percentile rank** | | **Immunogenicity score** | | **Antigenicity** |
| --- | --- | --- | --- | --- | --- | --- | --- | --- | --- | --- | --- |
| **DENV-1 NS1** | | | | | | | | | | | |
| 1 | 306 | 314 | | KTIHEWCCR | HLA-A*31:01 | 0.39 | | | 0.305 | | 0.5392 |
| 2 | 262 | 270 | | TQTAGPWHL | HLA-A*02:06 | 0.2 | | | 0.280 | | 0.9022 |
| 3 | 264 | 272 | | **TAGPWHLGK** | HLA-A*11:01 | 0.16 | | | 0.256 | | 1.0275 |
|  |  |  |  |  | HLA-A*03:01 | 0.41 | | |  |  |  |
| 4 | 155 | 163 | | VEDYGFGIF | HLA-B*40:01 | 0.32 | | | 0.253 | | 1.0582 |
| 5 | 331 | 339 | | YGMEIRPVK | HLA-A*30:01 | 0.4 | | | 0.237 | | 1.5242 |
| 6 | 152 | 160 | | IWEVEDYGF | HLA-A*24:02 | 0.3 | | | 0.209 | | 0.8882 |
|  |  |  |  |  | HLA-A*23:01 | 0.3 | | |  |  |  |
| 7 | 154 | 162 | | EVEDYGFGI | HLA-A*68:02 | 0.32 | | | 0.202 | | 1.6387 |
| 8 | 212 | 220 | | LARASFIEV | HLA-B*51:01 | 0.21 | | | 0.176 | | 0.6270 |
| 9 | 164 | 172 | | **TTNIWLKLR** | HLA-A*31:01 | 0.09 | | | 0.149 | | 0.8690 |
|  |  |  |  |  | HLA-A*33:01 | 0.11 | | |  |  |  |
|  |  |  |  |  | HLA-A*68:01 | 0.29 | | |  |  |  |
| 10 | 5 | 13 | | VINWKGREL | HLA-B*08:01 | 0.3 | | | 0.145 | | 1.9581 |
| 11 | 257 | 265 | | RPGYFTQTA | HLA-B*07:02 | 0.25 | | | 0.083 | | 1.4452 |
| 12 | 295 | 303 | | **GPSLRTTTV** | HLA-B*07:02 | 0.04 | | | 0.078 | | 1.1355 |
|  |  |  |  |  | HLA-B*08:01 | 0.16 | | |  |  |  |
|  |  |  |  |  | HLA-B*51:01 | 0.41 | | |  |  |  |
| 13 | 316 | 324 | | **CTLPPLRFK** | HLA-A*11:01 | 0.05 | | | 0.076 | | 1.5936 |
|  |  |  |  |  | HLA-A*03:01 | 0.06 | | |  |  |  |
|  |  |  |  |  | HLA-A*30:01 | 0.14 | | |  |  |  |
|  |  |  |  |  | HLA-A*31:01 | 0.41 | | |  |  |  |
|  |  |  |  |  | HLA-A*68:01 | 0.49 | | |  |  |  |
| 14 | 252 | 260 | | SQHNYRPGY | HLA-B*15:01 | 0.02 | | | 0.060 | | 1.4265 |
|  |  |  |  |  | HLA-A*30:02 | 0.08 | | |  |  |  |
| 15 | 303 | 311 | | VTGKTIHEW | HLA-A*32:01 | 0.05 | | | 0.043 | | 0.4322 |
|  |  |  |  |  | HLA-B*53:01 | 0.26 | | |  |  |  |
| 16 | 112 | 120 | | KYSWKSWGK | HLA-A*30:01 | 0.43 | | | 0.010 | | 0.9434 |
|  |  |  |  |  | HLA-A*31:01 | 0.45 | | |  |  |  |
| **DENV-3 NS1** | | | | | | | | | | | |
| 1 | 262 | 270 | TQTAGPWHL | | HLA-A*02:06 | | | 0.2 | | 0.280 | 0.9022 |
| 2 | 264 | 272 | TAGPWHLGK | | HLA-A*11:01 | | | 0.16 | | 0.256 | 1.0275 |
|  |  |  |  |  | HLA-A*03:01 | | | 0.41 | |  |  |
| 3 | 152 | 160 | VWEVEDYGF | | HLA-A*24:02 | | | 0.29 | | 0.209 | 0.9778 |
|  |  |  |  |  | HLA-A*23:01 | | | 0.31 | |  |  |
| 4 | 112 | 120 | KYSWKTWGK | | HLA-A*30:01 | | | 0.34 | | 0.202 | 0.4574 |
|  |  |  |  |  | HLA-A*31:01 | | | 0.35 | |  |  |
| 5 | 154 | 162 | EVEDYGFGV | | HLA-A*68:02 | | | 0.11 | | 0.202 | 1.5636 |
| 6 | 155 | 163 | VEDYGFGVF | | HLA-B*40:01 | | | 0.24 | | 0.199 | 1.0274 |
| 7 | 164 | 172 | **TTNIWLKLR** | | HLA-A*31:01 | | | 0.09 | | 0.149 | 0.8690 |
|  |  |  |  |  | HLA-A*33:01 | | | 0.11 | |  |  |
|  |  |  |  |  | HLA-A*68:01 | | | 0.29 | |  |  |
| 8 | 78 | 86 | **ILWENNIKL** | | HLA-A*02:01 | | | 0.01 | | 0.147 | 0.4510 |
|  |  |  |  |  | HLA-A*02:06 | | | 0.03 | |  |  |
|  |  |  |  |  | HLA-A*02:03 | | | 0.08 | |  |  |
|  |  |  |  |  | HLA-A*32:01 | | | 0.23 | |  |  |
| 9 | 70 | 78 | QIANELNYI | | HLA-A*02:06 | | | 0.5 | | 0.086 | 0.6535 |
| 10 | 257 | 265 | RPGYHTQTA | | HLA-B*07:02 | | | 0.1 | | 0.086 | 0.8350 |
| 11 | 295 | 303 | **GPSLRTTTV** | | HLA-B*07:02 | | | 0.04 | | 0.078 | 1.1355 |
|  |  |  |  |  | HLA-B*08:01 | | | 0.16 | |  |  |
|  |  |  |  |  | HLA-B*51:01 | | | 0.41 | |  |  |
| 12 | 252 | 260 | SQHNYRPGY | | HLA-B*15:01 | | | 0.02 | | 0.060 | 1.4265 |
|  |  |  |  |  | HLA-A*30:02 | | | 0.08 | |  |  |
| 13 | 316 | 324 | CTLPPLRYM | | HLA-B*57:01 | | | 0.38 | | 0.006 | 0.5426 |
| 14 | 315 | 323 | SCTLPPLRY | | HLA-A*30:02 | | | 0.22 | | 0.001 | 0.6635 |
|  |  |  |  |  | HLA-A*01:01 | | | 0.38 | |  |  |

**Supplementary Table 4.** Predicted MHC I epitopes for E protein of DENV-1 & 3 using IEDB server

| **Sl. No.** | **Start** | **End** | **Peptide** | **Allele** | **Percentile rank** | **Immunogenicity score** | **Anitigenicity** |
| --- | --- | --- | --- | --- | --- | --- | --- |
| **DENV-1** | | | | | | | |
| 1 | 417 | 425 | DTAWDFGSI | HLA-A*68:02 | 0.14 | 0.299 | 1.8803 |
| 2 | 154 | 162 | ETTEHGTTA | HLA-A*68:02 | 0.14 | 0.232 | 0.5484 |
| 3 | 38 | 46 | KPTLDIELL | HLA-B*53:01 | 0.35 | 0.226 | 0.6080 |
|  |  |  |  | HLA-B*07:02 | 0.5 |  |  |
| 4 | 136 | 144 | KYSVIVTVH | HLA-A*30:02 | 0.45 | 0.213 | 0.8546 |
| 5 | 457 | 465 | **IGIGILLTW** | HLA-B*58:01 | 0.04 | 0.210 | 1.5690 |
|  |  |  |  | HLA-B*57:01 | 0.06 |  |  |
|  |  |  |  | HLA-B*53:01 | 0.24 |  |  |
|  |  |  |  | HLA-A*32:01 | 0.25 |  |  |
| 6 | 353 | 361 | TANPIVTDK | HLA-A*11:01 | 0.14 | 0.201 | 0.7282 |
|  |  |  |  | HLA-A*68:01 | 0.19 |  |  |
| 7 | 403 | 411 | EATARGARR | HLA-A*33:01 | 0.16 | 0.198 | 0.7452 |
|  |  |  |  | HLA-A*68:01 | 0.32 |  |  |
| 8 | 262 | 270 | TALTGATEI | HLA-B*51:01 | 0.18 | 0.197 | 0.4863 |
| 9 | 228 | 236 | QETWNRQDL | HLA-B*40:01 | 0.25 | 0.193 | 0.5465 |
| 10 | 156 | 164 | TEHGTTATI | HLA-B*40:01 | 0.09 | 0.175 | 0.4220 |
|  |  |  |  | HLA-B*44:03 | 0.24 |  |  |
|  |  |  |  | HLA-B*44:02 | 0.27 |  |  |
| 11 | 412 | 420 | **MAILGDTAW** | HLA-B*53:01 | 0.02 | 0.142 | 0.7313 |
|  |  |  |  | HLA-B*58:01 | 0.04 |  |  |
|  |  |  |  | HLA-B*35:01 | 0.13 |  |  |
|  |  |  |  | HLA-B*57:01 | 0.15 |  |  |
| 12 | 160 | 168 | TTATITPQA | HLA-A*68:02 | 0.02 | 0.141 | 1.0907 |
| 13 | 424 | 432 | **SIGGVFTSV** | HLA-A*02:03 | 0.05 | 0.132 | 0.4735 |
|  |  |  |  | HLA-A*02:06 | 0.06 |  |  |
|  |  |  |  | HLA-A*02:01 | 0.13 |  |  |
|  |  |  |  | HLA-A*68:02 | 0.17 |  |  |
| 14 | 259 | 267 | AMHTALTGA | HLA-A*02:03 | 0.21 | 0.130 | 0.4295 |
| 15 | 233 | 241 | RQDLLVTFK | HLA-A*11:01 | 0.29 | 0.125 | 1.1671 |
|  |  |  |  | HLA-A*03:01 | 0.32 |  |  |
| 16 | 361 | 369 | KEKPVNIEA | HLA-B*40:01 | 0.46 | 0.124 | 1.3026 |
| 17 | 216 | 224 | LPLPWTSGA | HLA-B*51:01 | 0.47 | 0.118 | 1.0750 |
| 18 | 48 | 56 | TEVTNPAVL | HLA-B*40:01 | 0.02 | 0.093 | 0.5209 |
|  |  |  |  | HLA-B*44:03 | 0.34 |  |  |
|  |  |  |  | HLA-B*44:02 | 0.41 |  |  |
| 19 | 314 | 322 | ETQHGTVLV | HLA-A*68:02 | 0.01 | 0.093 | 0.5553 |
| 20 | 311 | 319 | EVAETQHGT | HLA-A*68:02 | 0.13 | 0.089 | 0.7871 |
| 21 | 170 | 178 | TSEIQLTDY | HLA-A*01:01 | 0.02 | 0.089 | 1.6089 |
| 22 | 190 | 198 | GLDFNEMVL | HLA-A*02:01 | 0.46 | 0.089 | 0.9508 |
| 23 | 175 | 183 | LTDYGALTL | HLA-A*01:01 | 0.22 | 0.087 | 1.0464 |
| 24 | 74 | 82 | CPTQGEATL | HLA-B*53:01 | 0.18 | 0.079 | 0.9870 |
|  |  |  |  | HLA-B*35:01 | 0.23 |  |  |
|  |  |  |  | HLA-B*07:02 | 0.28 |  |  |
| 25 | 447 | 455 | LFSGVSWTM | HLA-A*23:01 | 0.34 | 0.074 | 0.8300 |
|  |  |  |  | HLA-A*24:02 | 0.35 |  |  |
| 26 | 483 | 491 | GMVTLYLGV | HLA-A*02:03 | 0.49 | 0.049 | 0.6430 |
| 27 | 202 | 210 | KKKSWLVHK | HLA-A*30:01 | 0.16 | 0.023 | 1.0696 |
| 28 | 232 | 240 | NRQDLLVTF | HLA-A*23:01 | 0.46 | 0.021 | 1.0799 |
| 29 | 453 | 461 | WTMKIGIGI | HLA-A*68:02 | 0.22 | 0.020 | 1.3785 |
| 30 | 229 | 237 | ETWNRQDLL | HLA-A*68:02 | 0.15 | 0.019 | 0.6585 |
| 31 | 313 | 321 | AETQHGTVL | HLA-B*40:01 | 0.01 | 0.016 | 0.5192 |
|  |  |  |  | HLA-B*44:03 | 0.08 |  |  |
|  |  |  |  | HLA-B*44:02 | 0.08 |  |  |
| **DENV-3** | | | | | | | |
| 1 | 478 | 486 | **IAIGIITLY** | HLA-B*35:01 | 0.02 | 0.358 | 1.3468 |
|  |  |  |  | HLA-A*30:02 | 0.04 |  |  |
|  |  |  |  | HLA-A*26:01 | 0.05 |  |  |
|  |  |  |  | HLA-B*53:01 | 0.09 |  |  |
|  |  |  |  | HLA-B*58:01 | 0.1 |  |  |
|  |  |  |  | HLA-B*15:01 | 0.12 |  |  |
|  |  |  |  | HLA-B*57:01 | 0.18 |  |  |
|  |  |  |  | HLA-A*32:01 | 0.38 |  |  |
|  |  |  |  | HLA-A*01:01 | 0.43 |  |  |
| 2 | 415 | 423 | DTAWDFGSV | HLA-A*68:02 | 0.05 | 0.299 | 1.9031 |
| 3 | 313 | 321 | TQHGTILIK | HLA-A*30:01 | 0.18 | 0.276 | 0.9555 |
|  |  |  |  | HLA-A*11:01 | 0.26 |  |  |
|  |  |  |  | HLA-A*03:01 | 0.39 |  |  |
| 4 | 452 | 460 | VMKIGIGVL | HLA-B*08:01 | 0.42 | 0.275 | .8327 |
| 5 | 274 | 282 | TSIFAGHLK | HLA-A*11:01 | 0.09 | 0.252 | 0.5095 |
|  |  |  |  | HLA-A*68:01 | 0.2 |  |  |
| 6 | 372 | 380 | **GESNIVIGI** | HLA-B*40:01 | 0.1 | 0.240 | 0.9791 |
|  |  |  |  | HLA-B*44:03 | 0.18 |  |  |
|  |  |  |  | HLA-B*44:02 | 0.18 |  |  |
| 7 | 359 | 367 | KEEPVNIEA | HLA-B*40:01 | 0.47 | 0.226 | 1.1693 |
| 8 | 158 | 166 | VTAEITPQA | HLA-A*68:02 | 0.04 | 0.203 | 1.3999 |
|  |  |  |  | HLA-A*02:06 | 0.15 |  |  |
| 9 | 260 | 268 | TALTGATEI | HLA-B*51:01 | 0.18 | 0.197 | 0.4863 |
| 10 | 312 | 320 | ETQHGTILI | HLA-A*68:02 | 0.06 | 0.170 | 0.6346 |
| 11 | 39 | 47 | PTLDIELQK | HLA-A*11:01 | 0.43 | 0.166 | 0.8322 |
| 12 | 154 | 162 | ETQGVTAEI | HLA-A*68:02 | 0.01 | 0.165 | 0.4067 |
|  |  |  |  | HLA-A*26:01 | 0.21 |  |  |
| 13 | 226 | 234 | TPTWNRKEL | HLA-B*07:02 | 0.04 | 0.154 | 0.9820 |
| 14 | 384 | 392 | ALKINWYKK | HLA-A*30:01 | 0.31 | 0.137 | 0.8582 |
|  |  |  |  | HLA-A*03:01 | 0.46 |  |  |
| 15 | 257 | 265 | AMHTALTGA | HLA-A*02:03 | 0.21 | 0.130 | 0.4295 |
| 16 | 65 | 73 | ITNITTDSR | HLA-A*68:01 | 0.37 | 0.128 | 0.8680 |
| 17 | 455 | 463 | IGIGVLLTW | HLA-B*58:01 | 0.04 | 0.120 | 1.4585 |
|  |  |  |  | HLA-B*57:01 | 0.05 |  |  |
|  |  |  |  | HLA-B*53:01 | 0.22 |  |  |
|  |  |  |  | HLA-A*32:01 | 0.25 |  |  |
| 18 | 350 | 358 | **ITANPVVTK** | HLA-A*11:01 | 0.01 | 0.092 | 0.4101 |
|  |  |  |  | HLA-A*03:01 | 0.01 |  |  |
|  |  |  |  | HLA-A*30:01 | 0.04 |  |  |
|  |  |  |  | HLA-A*68:01 | 0.06 |  |  |
| 19 | 74 | 82 | CPTQGEAVL | HLA-B*35:01 | 0.23 | 0.080 | 0.7944 |
|  |  |  |  | HLA-B*53:01 | 0.32 |  |  |
|  |  |  |  | HLA-B*07:02 | 0.33 |  |  |
| 20 | 200 | 208 | KNKAWMVHR | HLA-A*31:01 | 0.04 | 0.074 | 0.7879 |
|  |  |  |  | HLA-A*33:01 | 0.41 |  |  |
| 21 | 311 | 319 | SETQHGTIL | HLA-B*40:01 | 0.01 | 0.070 | 0.4910 |
|  |  |  |  | HLA-B*44:02 | 0.14 |  |  |
|  |  |  |  | HLA-B*44:03 | 0.15 |  |  |
| 22 | 230 | 238 | NRKELLVTF | HLA-B*08:01 | 0.44 | 0.067 | 0.8414 |
| 23 | 316 | 324 | **GTILIKVEY** | HLA-A*30:02 | 0.11 | 0.052 | 1.4114 |
|  |  |  |  | HLA-A*26:01 | 0.2 |  |  |
|  |  |  |  | HLA-B*15:01 | 0.22 |  |  |
|  |  |  |  | HLA-A*32:01 | 0.33 |  |  |
|  |  |  |  | HLA-B*58:01 | 0.42 |  |  |
|  |  |  |  | HLA-A*11:01 | 0.47 |  |  |
|  |  |  |  | HLA-A*01:01 | 0.47 |  |  |
|  |  |  |  | HLA-B*57:01 | 0.49 |  |  |
| 24 | 317 | 325 | TILIKVEYK | HLA-A*11:01 | 0.16 | 0.042 | 1.7933 |
|  |  |  |  | HLA-A*03:01 | 0.49 |  |  |
| 25 | 309 | 317 | EVSETQHGT | HLA-A*68:02 | 0.26 | 0.023 | 0.8233 |
| 26 | 451 | 459 | WVMKIGIGV | HLA-A*68:02 | 0.21 | 0.020 | 0.9614 |

**Supplementary Table 5.** Predicted MHC II epitopes for NS1 protein of DENV-1 & 3 using IEDB server

| **S.**  **No** | **Peptide** | **Start** | | **End** | **Allele** | | **Percentile rank** | | **IFN-γ Score** |
| --- | --- | --- | --- | --- | --- | --- | --- | --- | --- |
| **DENV-1 NS1** | | | | | | | | | |
| 1 | SEKNETWKLARASFI | 204 | | 218 | HLA-DRB1*07:01 | | 0.1 | | 0.078 |
|  |  |  |  |  | HLA-DRB1*09:01 | | 0.45 | | 0.078 |
| 2 | DNQRAWNIWEVEDYG | 145 | | 159 | HLA-DQA1*01:01/DQB1*05:01 | | 0.17 | | 0.695 |
| 3 | PDNQRAWNIWEVEDY | 144 | | 158 | HLA-DQA1*01:01/DQB1*05:01 | | 0.17 | | 0.690 |
| 4 | NQRAWNIWEVEDYGF | 146 | | 160 | HLA-DQA1*01:01/DQB1*05:01 | | 0.2 | | 0.418 |
| 5 | **NETWKLARASFIEVK** | 207 | | 221 | HLA-DRB1*07:01 | | 0.21 | | 0.060 |
|  |  |  |  |  | HLA-DPA1*02:01/DPB1*14:01 | | 0.58 | |  |
|  |  |  |  |  | HLA-DRB1*09:01 | | 0.65 | |  |
|  |  |  |  |  | HLA-DRB3*02:02 | | 0.84 | |  |
|  |  |  |  |  | HLA-DRB5*01:01 | | 1.6 | |  |
|  |  |  |  |  | HLA-DPA1*01:03/DPB1*04:01 | | 2.3 | |  |
| 6 | QRAWNIWEVEDYGFG | 147 | | 161 | HLA-DQA1*01:01/DQB1*05:01 | | 0.28 | | 0.632 |
| 7 | RAWNIWEVEDYGFGI | 148 | | 162 | HLA-DQA1*01:01/DQB1*05:01 | | 0.33 | | 0.574 |
| 8 | **TWKLARASFIEVKTC** | 209 | | 223 | HLA-DRB1*07:01 | | 0.43 | | 0.200 |
|  |  |  |  |  | HLA-DPA1*02:01/DPB1*14:01 | | 0.7 | |  |
|  |  |  |  |  | HLA-DRB3*02:02 | | 1.9 | |  |
| 9 | **FGIFTTNIWLKLRDS** | 160 | | 174 | HLA-DPA1*01:03/DPB1*04:01 | | 0.52 | | 0.033 |
|  |  |  |  |  |  |  | 1.3 | |  |
|  |  |  |  |  |  |  | 2 | |  |
| 10 | ILAQGKKMIRPQPME | 96 | | 110 | HLA-DRB4*01:01 | | 0.69 | | 0.062 |
| 11 | GIFTTNIWLKLRDSY | 161 | | 175 | HLA-DPA1*01:03/DPB1*04:01 | | 1.1 | | 0.127 |
|  |  |  |  |  | HLA-DPA1*03:01/DPB1*04:02 | | 1.6 | |  |
| 12 | LNHILLENDMKFTVV | 75 | | 89 | HLA-DRB1*03:01 | | 1.1 | | 0.120 |
| 13 | ELNHILLENDMKFTV | 74 | | 88 | HLA-DRB1*03:01 | | 1.2 | | 0.211 |
| 14 | KAVHADMGYWIESEK | 192 | | 206 | HLA-DQA1*01:01/DQB1*05:01 | | 1.2 | | 0.054 |
|  |  |  |  |  | HLA-DQA1*03:01/DQB1*03:02 | | 2.3 | |  |
| 15 | NHILLENDMKFTVVV | 76 | | 90 | HLA-DRB1*03:01 | | 1.2 | | 0.313 |
| 16 | GSGIFVTNEVHTWTE | 16 | | 30 | HLA-DRB3*02:02 | | 1.4 | | 0.146 |
| 17 | SGIFVTNEVHTWTEQ | 17 | | 31 | HLA-DRB3*02:02 | | 1.4 | | 0.281 |
| 18 | HTLWSNGVLESEMII | 229 | | 243 | HLA-DQA1*03:01/DQB1*03:02 | | 1.7 | | 0.854 |
| 19 | GIFVTNEVHTWTEQY | 18 | | 32 | HLA-DRB3*02:02 | | 1.9 | | 0.470 |
| 20 | HILLENDMKFTVVVG | 77 | | 91 | HLA-DRB1*03:01 | | 2 | | 0.154 |
| 21 | ILLENDMKFTVVVGD | 78 | | 92 | HLA-DRB1*03:01 | | 2 | | 0.141 |
| 22 | GKLELDFDLCEGTTV | 271 | | 285 | HLA-DQA1*01:01/DQB1*05:01 | | 2.1 | | 0.065 |
| 23 | TLWSNGVLESEMIIP | 230 | | 244 | HLA-DQA1*03:01/DQB1*03:02 | | 2.1 | | 0.188 |
| 24 | PKSHTLWSNGVLESE | 226 | | 240 | HLA-DQA1*03:01/DQB1*03:02 | | 2.2 | | 0.318 |
| 25 | KSHTLWSNGVLESEM | 227 | | 241 | HLA-DQA1*03:01/DQB1*03:02 | | 2.3 | | 0.509 |
| 26 | AWNIWEVEDYGFGIF | 149 | | 163 | HLA-DQA1*01:01/DQB1*05:01 | | 2.4 | | 0.124 |
| 27 | DMKFTVVVGDVSGIL | 83 | | 97 | HLA-DRB1*03:01 | | 2.4 | | 0.111 |
| 28 | KFTVVVGDVSGILAQ | 85 | | 99 | HLA-DRB1*03:01 | | 2.4 | | 0.190 |
| 29 | MKFTVVVGDVSGILA | 84 | | 98 | HLA-DRB1*03:01 | | 2.4 | | 0.417 |
| 30 | FTVVVGDVSGILAQG | 86 | | 100 | HLA-DRB1*03:01 | | 2.5 | | 0.267 |
| **DENV-3 NS1** | | | | | | | | | |
| 1 | **FGVFTTNIWLKLREV** | 160 | 174 | | | HLA-DPA1*01:03/DPB1*04:01 | | 0.33 | 0.133 |
|  |  |  |  |  |  | HLA-DPA1*03:01/DPB1*04:02 | | 1.3 |  |
|  |  |  |  |  |  | HLA-DRB3*02:02 | | 1.7 |  |
| 2 | **YGFGVFTTNIWLKLR** | 158 | 172 | | | HLA-DPA1*01:03/DPB1*04:01 | | 0.33 | 0.109 |
|  |  |  |  |  |  | HLA-DPA1*03:01/DPB1*04:02 | | 1.4 |  |
|  |  |  |  |  |  | HLA-DRB3*02:02 | | 1.8 |  |
| 3 | GFGVFTTNIWLKLRE | 159 | 173 | | | HLA-DPA1*01:03/DPB1*04:01 | | 0.35 | 0.015 |
|  |  |  |  |  |  | HLA-DPA1*03:01/DPB1*04:02 | | 1.4 |  |
|  |  |  |  |  |  | HLA-DRB3*02:02 | | 1.8 |  |
| 4 | DYGFGVFTTNIWLKL | 157 | 171 | | | HLA-DPA1*01:03/DPB1*04:01 | | 0.48 | 0.104 |
|  |  |  |  |  |  | HLA-DPA1*03:01/DPB1*04:02 | | 1.4 |  |
| 5 | SRAWNVWEVEDYGFG | 147 | 161 | | | HLA-DQA1*01:01/DQB1*05:01 | | 0.56 | 0.092 |
| 6 | **GVFTTNIWLKLREVY** | 161 | 175 | | | HLA-DPA1*01:03/DPB1*04:01 | | 0.73 | 0.162 |
|  |  |  |  |  |  | HLA-DPA1*03:01/DPB1*04:02 | | 1.5 |  |
|  |  |  |  |  |  | HLA-DRB3*02:02 | | 2.4 |  |
| 7 | RAWNVWEVEDYGFGV | 148 | 162 | | | HLA-DQA1*01:01/DQB1*05:01 | | 0.74 | 0.366 |
| 8 | ELNYILWENNIKLTV | 74 | 88 | | | HLA-DRB1*13:02 | | 0.95 | 0.284 |
|  |  |  |  |  |  | HLA-DRB3*02:02 | | 2.2 |  |
| 9 | GSGIFVTNEVHTWTE | 16 | 30 | | | HLA-DRB3*02:02 | | 1.4 | 0.146 |
| 10 | NELNYILWENNIKLT | 73 | 87 | | | HLA-DRB1*13:02 | | 1.4 | 0.003 |
| 11 | RAVHADMGYWIESQK | 192 | 206 | | | HLA-DQA1*01:01/DQB1*05:01 | | 1.4 | 0.028 |
| 12 | SGIFVTNEVHTWTEQ | 17 | 31 | | | HLA-DRB3*02:02 | | 1.4 | 0.281 |
| 13 | GIFVTNEVHTWTEQY | 18 | 32 | | | HLA-DRB3*02:02 | | 1.9 | 0.470 |
| 14 | ERAVHADMGYWIESQ | 191 | 205 | | | HLA-DQA1*01:01/DQB1*05:01 | | 2.1 | 0.019 |

**Supplementary Table 6.** Predicted MHC II epitopes for E protein of DENV-1 & 3 using IEDB server

| **Sl. No.** | **Peptide** | **Start** | **End** | **Allele** | **Percentile rank** | **IFN-γ Score** |
| --- | --- | --- | --- | --- | --- | --- |
| **DENV-1 E** | | | | | | |
| 1 | LVHKQWFLDLPLPWT | 207 | 221 | HLA-DQA1*01:01/DQB1*05:01 | 0.01 | 0.091 |
|  |  |  |  | HLA-DPA1*01:03/DPB1*04:01 | 1.6 |  |
|  |  |  |  | HLA-DRB3*01:01 | 2.1 |  |
| 2 | **VHKQWFLDLPLPWTS** | 208 | 222 | HLA-DQA1*01:01/DQB1*05:01 | 0.02 | 0.072 |
|  |  |  |  | HLA-DRB3*01:01 | 2.1 |  |
|  |  |  |  | HLA-DPA1*01:03/DPB1*04:01 | 2.3 |  |
| 3 | GESYIVVGAGEKALK | 374 | 388 | HLA-DRB5*01:01 | 0.33 | 0.203 |
| 4 | **QDLLVTFKTAHAKKQ** | 234 | 248 | HLA-DRB1*04:01 | 0.41 | 0.308 |
|  |  |  |  | HLA-DRB1*15:01 | 0.64 |  |
|  |  |  |  | HLA-DRB5*01:01 | 0.85 |  |
|  |  |  |  | HLA-DRB1*08:02 | 0.59 |  |
| 5 | **DLLVTFKTAHAKKQE** | 235 | 249 | HLA-DRB1*04:01 | 0.42 | 0.143 |
|  |  |  |  | HLA-DRB1*08:02 | 0.64 |  |
|  |  |  |  | HLA-DRB1*15:01 | 0.73 |  |
|  |  |  |  | HLA-DRB5*01:01 | 0.77 |  |
| 6 | NEMVLLTMKKKSWLV | 194 | 208 | HLA-DRB1*11:01 | 0.48 | 0.544 |
| 7 | EMVLLTMKKKSWLVH | 195 | 209 | HLA-DRB1*11:01 | 0.52 | 0.533 |
| 8 | FNEMVLLTMKKKSWL | 193 | 207 | HLA-DRB1*11:01 | 0.52 | 0.210 |
|  |  |  |  | HLA-DRB5*01:01 | 1.9 |  |
| 9 | DFNEMVLLTMKKKSW | 192 | 206 | HLA-DRB1*11:01 | 0.54 | 0.336 |
|  |  |  |  | HLA-DRB5*01:01 | 1.9 |  |
| 10 | NRQDLLVTFKTAHAK | 232 | 246 | HLA-DRB1*04:01 | 0.55 | 0.265 |
|  |  |  |  | HLA-DRB1*08:02 | 0.61 |  |
| 11 | RQDLLVTFKTAHAKK | 233 | 247 | HLA-DRB1*04:01 | 0.55 | 0.356 |
|  |  |  |  | HLA-DRB1*08:02 | 0.59 |  |
|  |  |  |  | HLA-DRB1*15:01 | 0.64 |  |
| 12 | MVLLTMKKKSWLVHK | 196 | 210 | HLA-DRB1*11:01 | 0.59 | 0.540 |
| 13 | WNRQDLLVTFKTAHA | 231 | 245 | HLA-DRB1*04:01 | 0.59 | 0.204 |
|  |  |  |  | HLA-DRB1*08:02 | 0.6 |  |
|  |  |  |  | HLA-DRB1*15:01 | 0.72 |  |
| 14 | LLVTFKTAHAKKQEV | 236 | 250 | HLA-DRB5*01:01 | 0.63 | 0.245 |
| 15 | TLDIELLKTEVTNPA | 40 | 54 | HLA-DRB1*04:01 | 0.71 | 0.278 |
| 16 | LVTFKTAHAKKQEVV | 237 | 251 | HLA-DRB5*01:01 | 1.1 | 0.473 |
| 17 | GLDFNEMVLLTMKKK | 190 | 204 | HLA-DRB1*04:01 | 1.4 | 0.437 |
|  |  |  |  | HLA-DRB5*01:01 | 2 |  |
| 18 | CPTQGEATLVEEQDT | 74 | 88 | HLA-DQA1*03:01/DQB1*03:02 | 1.5 | 0.016 |
|  |  |  |  | HLA-DQA1*04:01/DQB1*04:02 | 1.6 |  |
| 19 | LDFNEMVLLTMKKKS | 191 | 205 | HLA-DRB1*04:01 | 1.5 | 0.255 |
|  |  |  |  | HLA-DRB5*01:01 | 1.8 |  |
|  |  |  |  | HLA-DRB1*11:01 | 2 |  |
| 20 | TGLDFNEMVLLTMKK | 189 | 203 | HLA-DRB1*04:01 | 1.5 | 0.070 |
| 21 | PTQGEATLVEEQDTN | 75 | 89 | HLA-DQA1*03:01/DQB1*03:02 | 1.6 | 0.015 |
|  |  |  |  | HLA-DQA1*04:01/DQB1*04:02 | 1.7 |  |
| 22 | RTGLDFNEMVLLTMK | 188 | 202 | HLA-DRB1*04:01 | 1.7 | 0.140 |
| 23 | GHLKCRLKMDKLILK | 281 | 295 | HLA-DRB1*03:01 | 2 | 0.399 |
| 24 | TALTGATEIQTSGTT | 262 | 276 | HLA-DQA1*01:02/DQB1*06:02 | 2.1 | 1.000 |
| 25 | VLLTMKKKSWLVHKQ | 197 | 211 | HLA-DRB1*11:01 | 2.2 | 0.741 |
| 26 | DYGALTLDCSPRTGL | 177 | 191 | HLA-DRB1*04:01 | 2.3 | 1.000 |
| 27 | GSIGGVFTSVGKLIH | 423 | 437 | HLA-DRB1*11:01 | 2.3 | 0.655 |
| 28 | IGGVFTSVGKLIHQI | 425 | 439 | HLA-DRB1*11:01 | 2.3 | 0.372 |
| 29 | QNGRLITANPIVTDK | 347 | 361 | HLA-DRB1*13:02 | 2.3 | 1.000 |
| 30 | SIGGVFTSVGKLIHQ | 424 | 438 | HLA-DRB1*11:01 | 2.3 | 0.505 |
| 31 | TQNGRLITANPIVTD | 346 | 360 | HLA-DRB1*13:02 | 2.3 | 0.079 |
| 32 | YGALTLDCSPRTGLD | 178 | 192 | HLA-DRB1*04:01 | 2.3 | 1.000 |
| 33 | YIVVGAGEKALKLSW | 377 | 391 | HLA-DRB5*01:01 | 2.3 | 1.000 |
| 34 | GALTLDCSPRTGLDF | 179 | 193 | HLA-DRB1*04:01 | 2.5 | 1.000 |
| 35 | KEKPVNIEAEPPFGE | 361 | 375 | HLA-DQA1*03:01/DQB1*03:02 | 2.5 | 0.052 |
| 36 | LLTWLGLNSRSTSLS | 462 | 476 | HLA-DRB1*01:01 | 2.5 | 0.017 |
| **DENV-3 E** | | | | | | |
| 1 | HRQWFFDLPLPWASG | 207 | 221 | HLA-DQA1*01:01/DQB1*05:01 | 0.01 | 0.429 |
|  |  |  |  | HLA-DRB3*01:01 | 2.4 |  |
| 2 | **MVHRQWFFDLPLPWA** | 205 | 219 | HLA-DQA1*01:01/DQB1*05:01 | 0.01 | 0.381 |
|  |  |  |  | HLA-DPA1*01:03/DPB1*04:01 | 1.4 |  |
|  |  |  |  | HLA-DRB3*01:01 | 2 | 0.260 |
| 3 | VHRQWFFDLPLPWAS | 206 | 220 | HLA-DQA1*01:01/DQB1*05:01 | 0.01 | 0.569 |
|  |  |  |  | HLA-DPA1*01:03/DPB1*04:01 | 1.7 |  |
|  |  |  |  | HLA-DRB3*01:01 | 2 |  |
| 4 | **WMVHRQWFFDLPLPW** | 204 | 218 | HLA-DQA1*01:01/DQB1*05:01 | 0.01 | 0.220 |
|  |  |  |  | HLA-DPA1*01:03/DPB1*04:01 | 1.4 |  |
|  |  |  |  | HLA-DPA1*01:03/DPB1*02:01 | 1.9 |  |
|  |  |  |  | HLA-DRB3*01:01 | 2 |  |
| 5 | KAWMVHRQWFFDLPL | 202 | 216 | HLA-DQA1*01:01/DQB1*05:01 | 0.02 | 0.219 |
|  |  |  |  | HLA-DPA1*01:03/DPB1*02:01 | 1.9 |  |
|  |  |  |  | HLA-DPA1*01:03/DPB1*04:01 | 2.3 |  |
| 6 | KELLVTFKNAHAKKQ | 232 | 246 | HLA-DRB1*15:01 | 0.14 | 0.169 |
|  |  |  |  | HLA-DRB1*04:01 | 0.36 |  |
|  |  |  |  | HLA-DRB1*08:02 | 0.45 |  |
|  |  |  |  | HLA-DRB5*01:01 | 1.8 |  |
| 7 | RKELLVTFKNAHAKK | 231 | 245 | HLA-DRB1*15:01 | 0.14 | 0.152 |
|  |  |  |  | HLA-DRB1*04:01 | 0.45 |  |
|  |  |  |  | HLA-DRB1*08:02 |  |  |
| 8 | **ELLVTFKNAHAKKQE** | 233 | 247 | HLA-DRB1*15:01 | 0.2 | 0.257 |
|  |  |  |  | HLA-DRB1*04:01 | 0.41 |  |
|  |  |  |  | HLA-DRB1*08:02 | 0.49 |  |
|  |  |  |  | HLA-DRB5*01:01 | 1.6 |  |
| 9 | RQWFFDLPLPWASGA | 208 | 222 | HLA-DQA1*01:01/DQB1*05:01 | 0.3 | 0.243 |
| 10 | EGKVVQYENLKYTVI | 126 | 140 | HLA-DRB1*15:01 | 0.36 | 0.059 |
| 11 | IELQKTEATQLATLR | 43 | 57 | HLA-DRB1*07:01 | 0.58 | 0.002 |
| 12 | NKAWMVHRQWFFDLP | 201 | 215 | HLA-DQA1*01:01/DQB1*05:01 | 0.6 | 0.027 |
|  |  |  |  | HLA-DPA1*01:03/DPB1*02:01 | 2 |  |
| 13 | PTLDIELQKTEATQL | 39 | 53 | HLA-DRB1*07:01 | 0.8 | 0.003 |
| 14 | HNGRLITANPVVTKK | 345 | 359 | HLA-DRB3*02:02 | 1.1 | 1.000 |
|  |  |  |  | HLA-DRB1*13:02 | 2.2 |  |
| 15 | NGRLITANPVVTKKE | 346 | 360 | HLA-DRB3*02:02 | 1.1 | 1.000 |
| 16 | LLVTFKNAHAKKQEV | 234 | 248 | HLA-DRB1*15:01 | 1.3 | 0.088 |
|  |  |  |  | HLA-DRB5*01:01 | 1.5 |  |
| 17 | GRLITANPVVTKKEE | 347 | 361 | HLA-DRB3*02:02 | 1.4 | 1.000 |
| 18 | LGKMVHQIFGSAYTA | 430 | 444 | HLA-DRB1*15:01 | 1.7 | 0.200 |
| 19 | GATEIQNSGGTSIFA | 264 | 278 | HLA-DRB1*13:02 | 1.8 | 2.000 |
| 20 | LVTFKNAHAKKQEVV | 235 | 249 | HLA-DRB1*15:01 | 1.8 | 0.260 |
|  |  |  |  | HLA-DRB5*01:01 | 2.3 |  |
| 21 | SLGKMVHQIFGSAYT | 429 | 443 | HLA-DRB1*15:01 | 1.8 | 0.153 |
| 22 | ATEIQNSGGTSIFAG | 265 | 279 | HLA-DRB1*13:02 | 1.9 | 2.000 |
| 23 | ILLTMKNKAWMVHRQ | 195 | 209 | HLA-DRB3*02:02 | 2 | 0.767 |
| 24 | KNKAWMVHRQWFFDL | 200 | 214 | HLA-DPA1*01:03/DPB1*02:01 | 2 | 0.296 |
| 25 | TGATEIQNSGGTSIF | 263 | 277 | HLA-DRB1*13:02 | 2 | 2.000 |
| 26 | RLITANPVVTKKEEP | 348 | 362 | HLA-DRB3*02:02 | 2.2 | 1.000 |
| 27 | EIQNSGGTSIFAGHL | 267 | 281 | HLA-DQA1*01:02/DQB1*06:02 | 2.3 | 2.000 |
| 28 | GSVGGVLNSLGKMVH | 421 | 435 | HLA-DRB1*11:01 | 2.3 | 0.092 |
| 29 | IQNSGGTSIFAGHLK | 268 | 282 | HLA-DQA1*01:02/DQB1*06:02 | 2.3 | 2.000 |
| 30 | TEIQNSGGTSIFAGH | 266 | 280 | HLA-DQA1*01:02/DQB1*06:02 | 2.4 | 2 |

| **DENV-1 NS1** | | | | **DENV-3 NS1** | | | |
| --- | --- | --- | --- | --- | --- | --- | --- |
| **Rank** | **Sequence** | **Start position** | **Score** | **Rank** | **Sequence** | **Start position** | **Score** |
| 1 | **SQHNYRPGYFTQTAGP** | 252 | 0.96 | 1 | SQHNYRPGYHTQTAGP | 252 | 0.94 |
| 1 | TFIIDGPNTPECPDNQ | 132 | 0.96 | 2 | **SFIIDGPNTPECPSAS** | 132 | 0.93 |
| 2 | **YGMEIRPVKEKEENLV** | 331 | 0.9 | 3 | VCGIRSTTRMENLLWK | 54 | 0.88 |
| 3 | GKTIHEWCCRSCTLPP | 305 | 0.89 | 3 | **GKLIHEWCCRSCTLPP** | 305 | 0.88 |
| 3 | AVHADMGYWIESEKNE | 193 | 0.89 | 3 | PNTPECPSASRAWNVW | 138 | 0.88 |
| 4 | IWEVEDYGFGIFTTNI | 152 | 0.87 | 4 | YHTQTAGPWHLGKLEL | 260 | 0.87 |
| 5 | CGIRSATRLENIMWKQ | 55 | 0.85 | 4 | TQLCDHRLMSAAVKDE | 176 | 0.87 |
| 5 | YFTQTAGPWHLGKLEL | 260 | 0.85 | 4 | VWEVEDYGFGVFTTNI | 152 | 0.87 |
| 6 | VHTWTEQYKFQADSPK | 25 | 0.84 | 5 | AVHADMGYWIESQKNG | 193 | 0.85 |
| 7 | RFKGEDGCWYGMEIRP | 322 | 0.83 | 6 | VHTWTEQYKFQADSPK | 25 | 0.84 |
| 7 | YTQVCDHRLMSAAIKD | 175 | 0.83 | 6 | ELKYSWKTWGKAKIVT | 110 | 0.84 |
| 8 | CRSCTLPPLRFKGEDG | 313 | 0.82 | 7 | LATAIAGAWENGVCGI | 42 | 0.83 |
| 9 | GKAWEEGVCGIRSATR | 47 | 0.81 | 8 | GCWYGMEIRPISEKEE | 328 | 0.82 |
| 9 | TVVVDEHCGNRGPSLR | 284 | 0.81 | 8 | VVITESCGTRGPSLRT | 285 | 0.82 |
| 9 | GFGIFTTNIWLKLRDS | 159 | 0.81 | 9 | RYMGEDGCWYGMEIRP | 322 | 0.81 |
| 9 | KKMIRPQPMEHKYSWK | 101 | 0.81 | 10 | GVLESDMIIPKSLAGP | 235 | 0.8 |
| 10 | KSWGKAKIIGADVQNT | 116 | 0.8 | 10 | HTLWTNGVLESDMIIP | 229 | 0.8 |

**Supplementary Table 7.** Predicted B Cell epitopes for NS1 protein of DENV-1 & 3 through ABCPred server

**Supplementary Table 8.** Predicted B Cell epitopes for E protein of DENV-1 & 3 through ABCPred server

| **DENV-1 E** | | | | **DENV-3 E** | | | |
| --- | --- | --- | --- | --- | --- | --- | --- |
| **Rank** | **Sequence** | **Start position** | **Score** | **Rank** | **Sequence** | **Start position** | **Score** |
| 1 | **HQVGNETTEHGTTATI** | 149 | 0.92 | 1 | **DGQGKAHNGRLITANP** | 339 | 0.94 |
| 2 | **AILGDTAWDFGSIGGV** | 413 | 0.91 | 1 | **HGTILIKVEYKGEDAP** | 315 | 0.94 |
| 3 | GVSWTMKIGIGILLTW | 450 | 0.9 | 1 | GATTETPTWNRKELLV | 221 | 0.94 |
| 3 | DCSPRTGLDFNEMVLL | 184 | 0.9 | 2 | GRLITANPVVTKKEEP | 347 | 0.93 |
| 4 | TTMAKDKPTLDIELLK | 32 | 0.89 | 3 | TTMAKNKPTLDIELQK | 32 | 0.9 |
| 5 | PVNIEAEPPFGESYIV | 364 | 0.87 | 3 | YGTLGLECSPRTGLDF | 176 | 0.9 |
| 6 | YGALTLDCSPRTGLDF | 178 | 0.86 | 3 | HQVGNETQGVTAEITP | 149 | 0.9 |
| 7 | RGWGNGCGLFGKGSLI | 99 | 0.85 | 4 | PVNIEAEPPFGESNIV | 362 | 0.88 |
| 7 | STSQETWNRQDLLVTF | 225 | 0.85 | 4 | KGMSYAMCTNTFVLKK | 293 | 0.88 |
| 7 | TSEIQLTDYGALTLDC | 170 | 0.85 | 5 | ECSPRTGLDFNEMILL | 182 | 0.87 |
| 7 | IVTVHTGDQHQVGNET | 140 | 0.85 | 6 | YVCKHTYVDRGWGNGC | 90 | 0.86 |
| 8 | PCKIPFSSQDEKGVTQ | 332 | 0.84 | 7 | RGWGNGCGLFGKGSLV | 99 | 0.85 |
| 9 | KGMSYVMCTGSFKLEK | 295 | 0.83 | 7 | GVSWVMKIGIGVLLTW | 448 | 0.85 |
| 10 | PTQGEATLVEEQDTNF | 75 | 0.81 | 7 | TQGVTAEITPQASTTE | 155 | 0.85 |
| 10 | TTDSRCPTQGEATLVE | 69 | 0.81 | 8 | IGKMFEATERGARRMA | 396 | 0.83 |
| 10 | ATEIQTSGTTTIFAGH | 267 | 0.81 | 8 | GGTSIFAGHLKCRLKM | 272 | 0.83 |
| 11 | FVCRRTFVDRGWGNGC | 90 | 0.8 | 9 | CPTQGEAVLPEEQDQN | 74 | 0.82 |
| 11 | YEGTDAPCKIPFSSQD | 326 | 0.8 | 9 | AILGDTAWDFGSVGGV | 411 | 0.82 |
| 11 | AGHLKCRLKMDKLILK | 280 | 0.8 | 9 | AGHLKCRLKMDKLELK | 278 | 0.82 |
| 11 | RCVGIGNRDFVEGLSG | 2 | 0.8 | 10 | AWMVHRQWFFDLPLPW | 203 | 0.81 |
| 11 | TITPQAPTSEIQLTDY | 163 | 0.8 | 10 | TVIITVHTGDQHQVGN | 138 | 0.81 |
| 11 | KIVQYENLKYSVIVTV | 128 | 0.8 | 10 | KVVQYENLKYTVIITV | 128 | 0.81 |
|  |  |  |  | 11 | GAMHTALTGATEIQNS | 256 | 0.8 |

**Supplementary Table 9:** Predicted Discontinuous Epitopes in the vaccine construct.

| **S. No.** | **Residues** | **Number of residues** | **Score** |
| --- | --- | --- | --- |
| 1 | A:H560, A:G561, A:T562, A:I563, A:L564, A:I565, A:K566, A:V567, A:E568, A:Y569, A:K570, A:G571, A:E572, A:D573, A:A574, A:P575 | 16 | 0.911 |
| 2 | A:K179, A:Y182, A:G183, A:T184, A:I185, A:L186, A:I187, A:K188, A:V189, A:E190, A:Y191, A:G192, A:P193, A:G194, A:P195, A:G196, A:N197, A:E198, A:T199, A:W200, A:K201, A:L202, A:A203, A:R204, A:A205, A:S206, A:F207, A:I208, A:E209, A:V210, A:K211, A:G212, A:P213, A:G214, A:P215, A:G216, A:T217, A:W218, A:K219, A:L220, A:A221, A:R222, A:A223, A:S224, A:F225, A:I226, A:E227, A:V228, A:K229, A:T230, A:C231, A:G232, A:P326, A:P328, A:W329, A:T330, A:S331, A:G332, A:P333, A:G334, A:P335, A:G336, A:Q337, A:D338, A:L339, A:L340, A:V341, A:T342, A:F343, A:K344, A:T345, A:A346, A:H347, A:A348, A:K349, A:K350, A:Q351, A:G352, A:P353, A:G354, A:P355, A:G356 | 82 | 0.819 |
| 3 | A:G31, A:S34, A:R36, A:G37, A:R38, A:K39, A:C40, A:C41, A:R42, A:R43, A:K44, A:K45, A:E46, A:A47, A:A48, A:A49, A:K50, A:T51, A:A52, A:K59, A:I136, A:G137, A:G138, A:V139, A:F140, A:T141, A:S142, A:A157, A:G159, A:E160, A:S161, A:F240, A:T241, A:T242, A:N243, A:P253, A:G254, A:P255, A:G256, A:F257, A:G258, A:V259, A:F260, A:T261, A:T262, A:N263, A:W265, A:L266, A:L268, A:R269, A:E270, A:V271, A:G272, A:P273, A:G274, A:P275, A:G276, A:Y277, A:G278, A:F279, A:G280, A:V281, A:F282, A:T283, A:W287, A:L288, A:L290, A:R291, A:G292, A:P293, A:G294, A:P387, A:L388, A:P389, A:W390, A:A391, A:G392, A:P393, A:G394, A:P395, A:G396, A:W397, A:M398, A:V399, A:H400, A:R401, A:Q402, A:W403, A:F404, A:F405, A:D406, A:L407, A:P408, A:L409, A:P410, A:F422, A:K423, A:N424, A:A425, A:H426, A:A427, A:K428, A:K429, A:Q430, A:E431, A:K432, A:K433, A:S434, A:Q435, A:H436, A:N437, A:Y438, A:R439, A:P440, A:G441, A:Y442, A:F443, A:T444, A:Q445, A:T446, A:A447, A:G448, A:P449, A:K450, A:K451, A:G457, A:P458, A:N459, A:T460, A:P461, A:E462, A:C463, A:P464, A:S465, A:A466, A:S467, A:K468, A:K469, A:V477, A:K478, A:E479, A:K480, A:E481, A:E482, A:L484, A:V485, A:K486, A:K487, A:G488, A:K489, A:L490, A:I491, A:H492, A:E493, A:C495, A:C496, A:S498, A:C499, A:T500, A:L501, A:G534, A:S535, A:I536, A:G537, A:G538, A:V539, A:K540, A:K541, A:D542, A:G543, A:Q544, A:G545, A:K546, A:A547, A:H548 | 175 | 0.655 |
| 4 | A:G1, A:I2, A:I3, A:N4, A:T5, A:L6, A:Q7, A:K8, A:Y9, A:Y10, A:C11, A:R12, A:V13, A:R14, A:G15, A:G16, A:R17, A:C18, A:A19, A:V20, A:L21, A:S22, A:L24, A:P25, A:K26, A:E27, A:E28, A:P54, A:G58, A:A60, A:A61, A:Y62, A:T63, A:T64, A:N65, A:K69, A:L92, A:R93, A:F94, A:K95, A:A96, A:A97, A:Y98 | 43 | 0.558 |
| 5 | A:N549, A:G550, A:R551 | 3 | 0.546 |
| 6 | A:E309, A:V310, A:Y311 | 3 | 0.519 |
| 7 | A:E371, A:G372, A:P373 | 3 | 0.501 |

**Supplementary Table 10:** List of residue pairs in the vaccine construct that have the ability to create disulfide bonds.

| **Res1 Chain** | **Res1 Seq #** | **Res1 AA** | **Res2 Chain** | **Res2 Seq #** | **Res2 AA** | **Chi3** | **Energy** | **Sum B-Factors** |
| --- | --- | --- | --- | --- | --- | --- | --- | --- |
| A | 1 | GLY | A | 5 | THR | 98.17 | 2.7 | 0 |
| A | 1 | GLY | A | 22 | SER | 107.2 | 2.9 | 0 |
| A | 2 | ILE | A | 22 | SER | -88.47 | 3.78 | 0 |
| A | 3 | ILE | A | 16 | GLY | 82.56 | 7.73 | 0 |
| A | 6 | LEU | A | 92 | LEU | 119.07 | 3.05 | 0 |
| A | 7 | GLN | A | 13 | VAL | 84.31 | 6.1 | 0 |
| A | 8 | LYS | A | 18 | CYS | -67.42 | 5.66 | 0 |
| A | 9 | TYR | A | 54 | PRO | 105.65 | 4.69 | 0 |
| A | 10 | TYR | A | 54 | PRO | 106.07 | 3.68 | 0 |
| A | 23 | CYS | A | 100 | LEU | 83.58 | 4.46 | 0 |
| A | 24 | LEU | A | 26 | LYS | -83.64 | 3.85 | 0 |
| A | 25 | PRO | A | 106 | LYS | 97.97 | 0.61 | 0 |
| A | 28 | GLU | A | 61 | ALA | -75.23 | 2.81 | 0 |
| A | 30 | ILE | A | 55 | TRP | -115 | 2.51 | 0 |
| A | 30 | ILE | A | 57 | LEU | -109.95 | 2.82 | 0 |
| A | 31 | GLY | A | 51 | THR | -105.67 | 4.58 | 0 |
| A | 32 | LYS | A | 35 | THR | 104.27 | 5.02 | 0 |
| A | 34 | SER | A | 40 | CYS | -91.03 | 2.01 | 0 |
| A | 35 | THR | A | 53 | GLY | 85.19 | 4.94 | 0 |
| A | 36 | ARG | A | 46 | GLU | 104.51 | 3.13 | 0 |
| A | 38 | ARG | A | 45 | LYS | -80.82 | 3.94 | 0 |
| A | 38 | ARG | A | 46 | GLU | 117.24 | 9.09 | 0 |
| A | 41 | CYS | A | 501 | LEU | 118.46 | 2.69 | 0 |
| A | 42 | ARG | A | 44 | LYS | 112.59 | 0.88 | 0 |
| A | 43 | ARG | A | 49 | ALA | -115.74 | 5.39 | 0 |
| A | 47 | ALA | A | 51 | THR | -91.76 | 1 | 0 |
| A | 48 | ALA | A | 51 | THR | 108.69 | 1.76 | 0 |
| A | 55 | TRP | A | 87 | CYS | 84.33 | 4.79 | 0 |
| A | 57 | LEU | A | 106 | LYS | 82.24 | 3.41 | 0 |
| A | 75 | GLY | A | 363 | LYS | 71.68 | 5.08 | 0 |
| A | 77 | SER | A | 557 | PRO | 104.77 | 3.31 | 0 |
| A | 80 | THR | A | 364 | THR | -79.43 | 2.3 | 0 |
| A | 84 | ALA | A | 107 | LEU | 84.39 | 5.06 | 0 |
| A | 85 | ALA | A | 89 | LEU | 89.5 | 3.8 | 0 |
| A | 85 | ALA | A | 109 | ALA | 77.28 | 5.15 | 0 |
| A | 90 | PRO | A | 99 | ILE | 68.56 | 7.17 | 0 |
| A | 90 | PRO | A | 101 | TRP | 107.12 | 2.65 | 0 |
| A | 91 | PRO | A | 93 | ARG | -87.03 | 5.93 | 0 |
| A | 101 | TRP | A | 109 | ALA | -61.64 | 5.11 | 0 |
| A | 110 | TYR | A | 150 | GLY | -102.88 | 4.17 | 0 |
| A | 116 | LEU | A | 119 | TRP | -93.01 | 5.21 | 0 |
| A | 118 | THR | A | 121 | ALA | 99.28 | 3.19 | 0 |
| A | 123 | MET | A | 252 | GLY | -82.36 | 2.92 | 0 |
| A | 125 | ILE | A | 164 | VAL | 122.03 | 5.5 | 0 |
| A | 141 | THR | A | 388 | LEU | -106.46 | 5.7 | 0 |
| A | 145 | ALA | A | 384 | PHE | -111.07 | 6.01 | 0 |
| A | 152 | ILE | A | 166 | GLY | -90.52 | 2.28 | 0 |
| A | 161 | SER | A | 392 | GLY | -88.86 | 5.03 | 0 |
| A | 169 | ALA | A | 247 | LYS | 106.64 | 3.87 | 0 |
| A | 170 | TYR | A | 377 | MET | -93.33 | 4.86 | 0 |
| A | 173 | ALA | A | 243 | ASN | -100.37 | 5.05 | 0 |
| A | 175 | PRO | A | 237 | PHE | 95.85 | 3.34 | 0 |
| A | 178 | THR | A | 236 | GLY | -108.86 | 5.21 | 0 |
| A | 181 | ALA | A | 234 | GLY | 124.63 | 7.02 | 0 |
| A | 183 | GLY | A | 232 | GLY | -93.99 | 7.16 | 0 |
| A | 186 | LEU | A | 229 | LYS | 123.93 | 8.12 | 0 |
| A | 188 | LYS | A | 227 | GLU | -97.09 | 7.69 | 0 |
| A | 192 | GLY | A | 223 | ALA | -73.56 | 1.45 | 0 |
| A | 199 | THR | A | 201 | LYS | -107.58 | 3.88 | 0 |
| A | 201 | LYS | A | 225 | PHE | -68.16 | 0.97 | 0 |
| A | 214 | GLY | A | 222 | ARG | 106.04 | 2.59 | 0 |
| A | 216 | GLY | A | 220 | LEU | 97.91 | 1.04 | 0 |
| A | 241 | THR | A | 289 | LYS | 122.23 | 3.06 | 0 |
| A | 248 | LEU | A | 266 | LEU | 118.47 | 7.46 | 0 |
| A | 249 | ARG | A | 252 | GLY | 75.14 | 4.59 | 0 |
| A | 256 | GLY | A | 260 | PHE | -72.56 | 3.72 | 0 |
| A | 275 | PRO | A | 284 | THR | -111.8 | 2.16 | 0 |
| A | 287 | TRP | A | 292 | GLY | 83.23 | 5.5 | 0 |
| A | 287 | TRP | A | 383 | PHE | 82.54 | 3.74 | 0 |
| A | 308 | ARG | A | 311 | TYR | 125.51 | 9.25 | 0 |
| A | 315 | PRO | A | 365 | ALA | 107.55 | 2.83 | 0 |
| A | 344 | LYS | A | 352 | GLY | -103.99 | 8.7 | 0 |
| A | 394 | GLY | A | 460 | THR | 125.75 | 4.34 | 0 |
| A | 400 | HIS | A | 431 | GLU | 124.92 | 2.95 | 0 |
| A | 401 | ARG | A | 426 | HIS | 74.87 | 4.65 | 0 |
| A | 403 | TRP | A | 425 | ALA | -75.64 | 4.33 | 0 |
| A | 405 | PHE | A | 423 | LYS | -98.78 | 6.31 | 0 |
| A | 427 | ALA | A | 466 | ALA | -61.95 | 4.31 | 0 |
| A | 433 | LYS | A | 458 | PRO | -85.04 | 2.96 | 0 |
| A | 436 | HIS | A | 449 | PRO | 104.3 | 3.61 | 0 |
| A | 437 | ASN | A | 451 | LYS | -92.66 | 4.13 | 0 |
| A | 440 | PRO | A | 443 | PHE | 120.67 | 4.42 | 0 |
| A | 478 | LYS | A | 499 | CYS | 101.53 | 1.43 | 0 |
| A | 498 | SER | A | 502 | PRO | 86.26 | 4.27 | 0 |
| A | 526 | LEU | A | 530 | ALA | 107.48 | 4.39 | 0 |
| A | 531 | TRP | A | 545 | GLY | 97.38 | 2.53 | 0 |
| A | 534 | GLY | A | 541 | LYS | 95.08 | 2.24 | 0 |
| A | 538 | GLY | A | 541 | LYS | 82.56 | 4.92 | 0 |
| A | 560 | HIS | A | 564 | LEU | 116.85 | 3.81 | 0 |

**
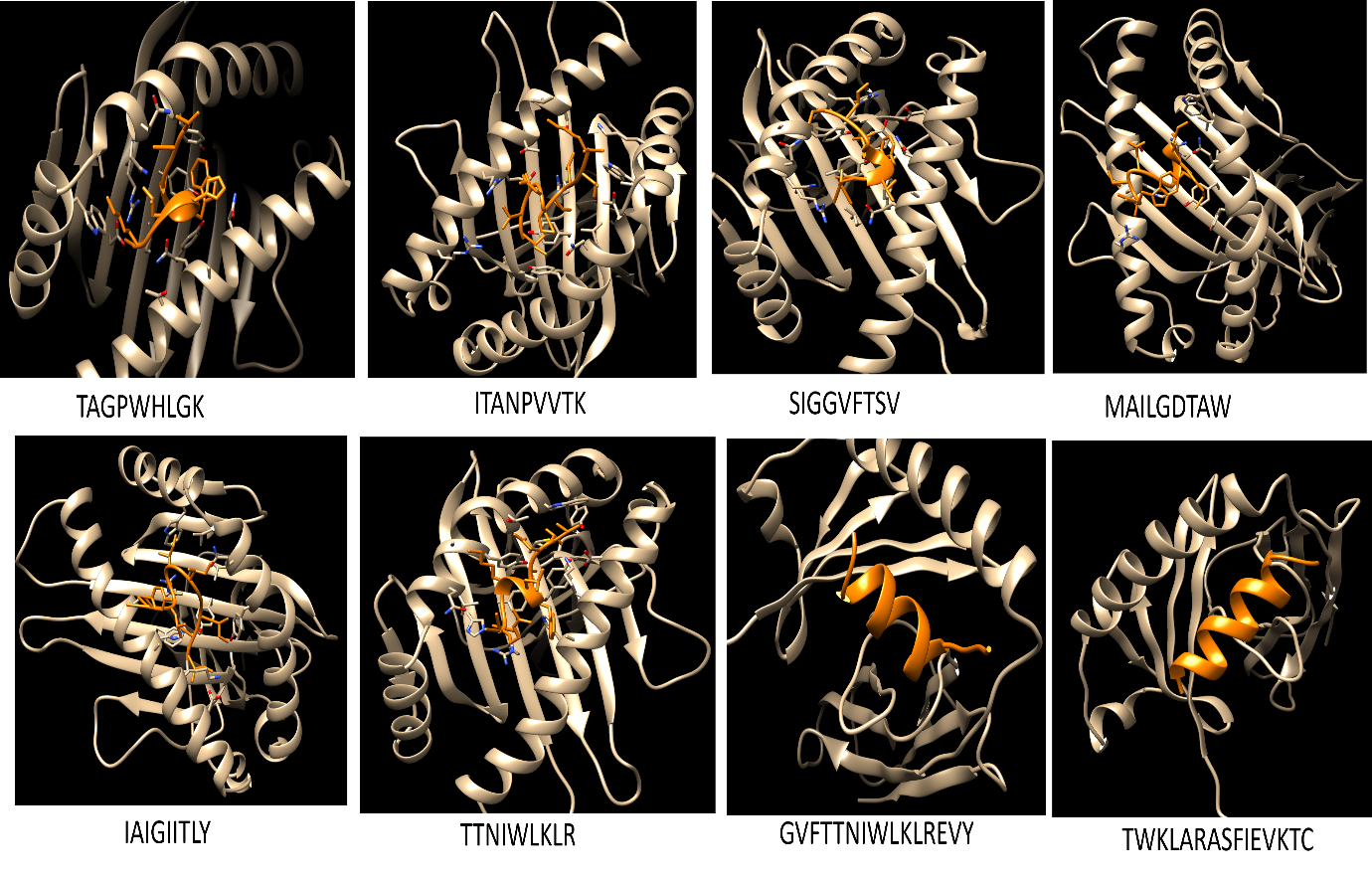
****Supplementary Fig. S1:** Representative PatchDock models of selected T-cell epitope–HLA complexes.


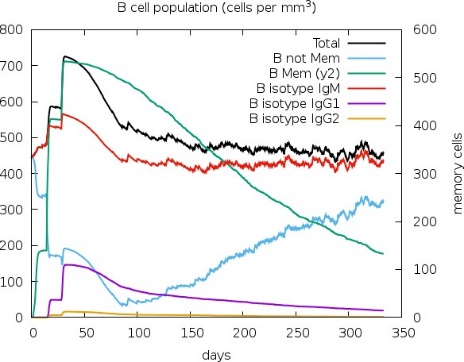

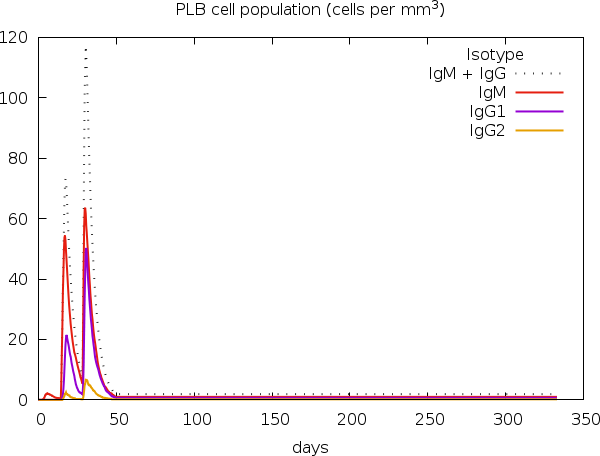

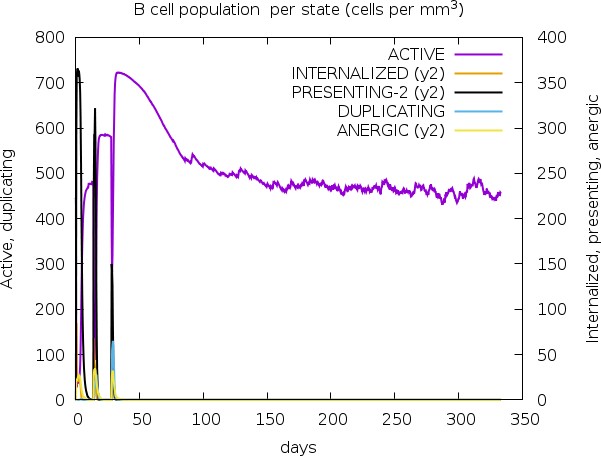

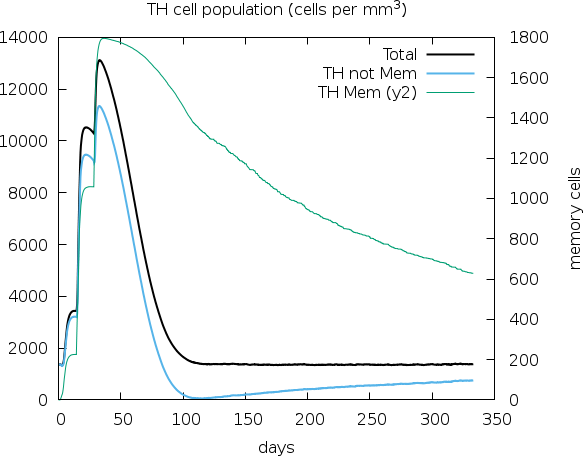

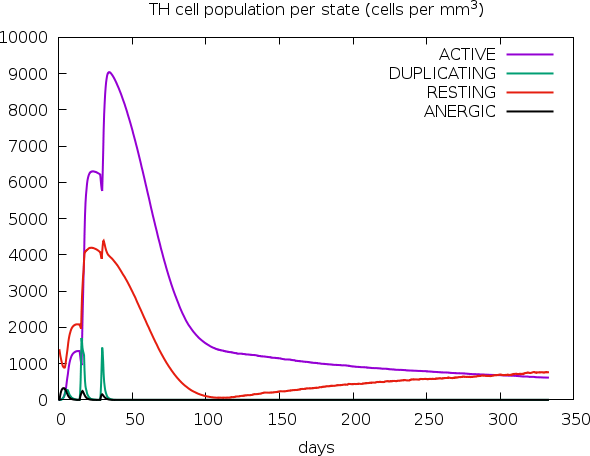

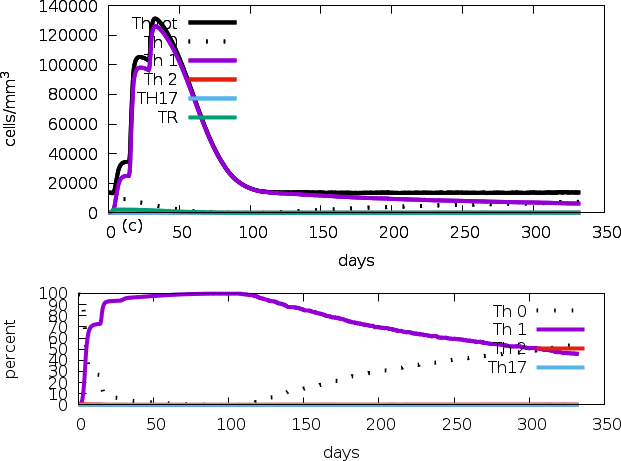


**Supplementary Fig. S2.** C-IMMSIM simulation result (Cell counts shown. Legend: Act=active, Intern=internalized the Ag, Pres II = presenting on MHC II, Dup=in the mitotic cycle, Anergic=anergic, Resting=not active).


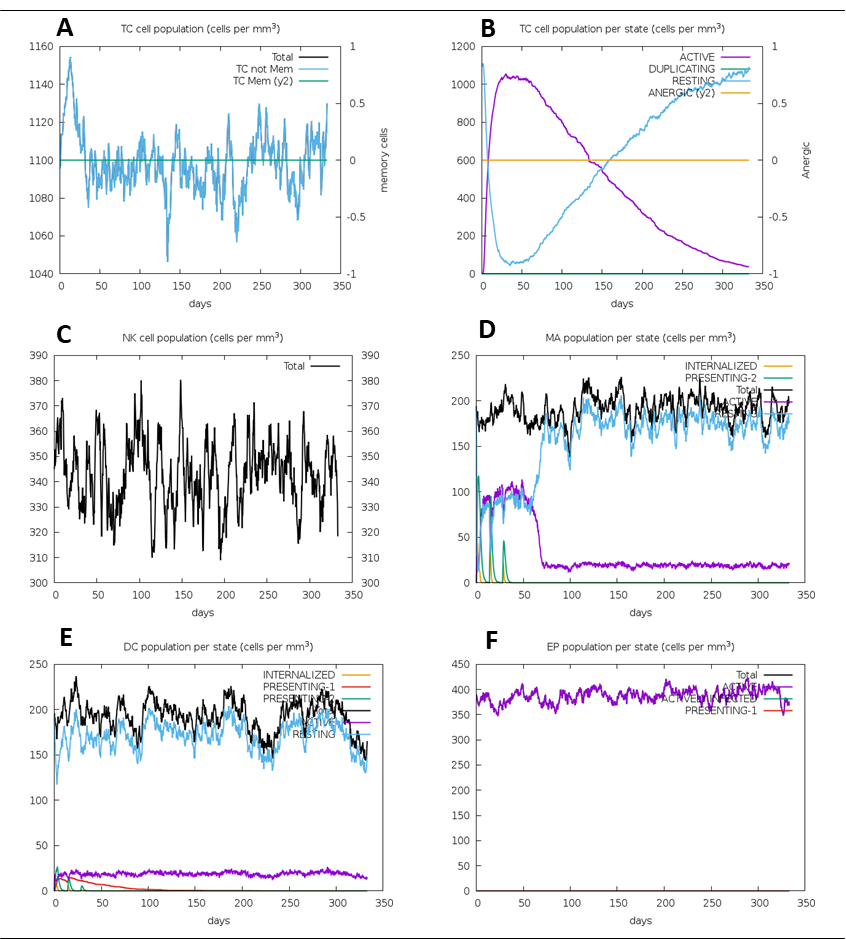


**Supplementary Fig. S3**. C-IMMSIM simulation result (A) TC cell population, (B) TC cell population per state, (C) NK cell population, (D) NK cell population per state, (E) DC population per state, (F) EP population per state.
